# WHO Environmental Noise Guidelines for the European Region: A Systematic Review on Environmental Noise and Quality of Life, Wellbeing and Mental Health

**DOI:** 10.3390/ijerph15112400

**Published:** 2018-10-29

**Authors:** Charlotte Clark, Katarina Paunovic

**Affiliations:** 1 Acoustics, Ove Arup & Partners, 13 Fitzroy Street, London W1T 4BQ, UK; 2 Institute of Hygiene and Medical Ecology, Faculty of Medicine, University of Belgrade, Belgrade 11000, Serbia; paunkaya@yahoo.com

**Keywords:** road traffic noise, aircraft noise, railway noise, wind-turbine noise, quality of life, wellbeing, depression, anxiety, mental health

## Abstract

This systematic review assesses the quality of the evidence across studies on the effect of environmental noise (road traffic noise, aircraft noise, railway noise, wind-turbine noise) on quality of life, wellbeing and mental health. Quantitative studies of noise effects on children and adults published from January 2005 up to October 2015 were reviewed. A total of 29 papers were identified. 90% of the papers were of cross-sectional design, with fewer studies of longitudinal or intervention design. Outcomes included depression and anxiety, medication use and childhood emotional problems. The quality of the evidence across the studies for each individual noise source was assessed using an adaptation of the GRADE methodology. Overall, given the predominance of cross-sectional studies, most evidence was rated as very low quality, with evidence of effects only being observed for some noise sources and outcomes. These ratings reflect inconsistent findings across studies, the small number of studies and a lack of methodological robustness within some domains. Overall, there are few studies of clinically significant mental health outcomes; few studies of railway noise exposure; and studies of large samples are needed. The lack of evidence for noise effects across studies for many of the quality of life, wellbeing and mental health domains examined does not necessarily mean that there are no effects: rather, that they have not yet been studied robustly for different noise sources.

## 1. Introduction

This paper is a systematic review of evidence for effects of environmental noise on quality of life, wellbeing and mental health. This paper is the sister paper to the previously published evidence review on the effects of environmental noise on cognition [1]. Both reviews were undertaken at the same time, using the same methodology, to inform the World Health Organization’s revision of their Guidelines for Community Noise [2]. The existing WHO guidelines cover exposure in both home and school environments: both contexts that have been the focus of studies on noise effects on mental health in children and adult populations. Within the home environment, the previous WHO Community Noise Guidelines specify that the background sound pressure should not exceed 50 dB L_Aeq 16 hour_ in outdoor living areas in the day-time and evening and that levels should not exceed 30 dB L_Aeq 8 hour_ outside bedrooms. 

Several plausible pathways and mechanisms for the effects of environmental noise on quality of life, wellbeing and mental health have been put forward. Noise is thought to be an environmental stressor and effects on quality of life, wellbeing and mental health are thought to operate via the stress-diathesis hypothesis [3,4,5]. Acute noise exposure results in increased physiological arousal via stimulation of the endocrine system and autonomic nervous system [2], which leads to an increase in stress hormones like catecholamines (e.g., adrenaline/noradrenaline) and cortisol. Chronic noise exposure may cause prolonged activation of these responses, which can lead to the development of depression and anxiety disorders [6,7,8]. Psychological stress responses might also be implicated in low mood, such as annoyance, which may directly activate physiological stress hormones. 

## 2. Materials and Methods

### Scope of the Review

The review used the term mental health to refer to a range of mental health symptoms and diagnoses that might be indicative of moderate to severe mental ill-health such as depressive episodes or anxiety disorder in adults, and emotional disorders, conduct disorder, and hyperactivity in children. Mental health is often viewed as on a continuum whereby symptoms precede more serious clinically significant diagnoses that can be made using standardized diagnostic criteria such as the ICD (International Classification of Diseases) [9] and DSM (Diagnostic Statistical Manual) [10]. The review additionally considers studies that examine the use of psychotropic medication such as anti-depressants and anti-anxiety medication. The review also examines the quality of the evidence for noise effects on quality of life and self-reported health within its remit.

Search terms covering different sources of environmental noise (aircraft, road traffic, railway, wind-turbine), different study designs (cross-sectional, longitudinal), and different quality of life, wellbeing and mental health outcomes (self-reported quality of life; health-related quality of life; medication intake for treatment of anxiety and depression; self-reported depression, anxiety and psychological symptoms; interview measures of depressive and anxiety disorders; hospital admission data for psychiatric disorders; emotional and conduct disorders in children) were included in database searches of Medline/Pubmed; Scopus (includes Embase); PsycInfo, Web of Science Database and ScienceDirect. Due to time constraints, conference proceedings for ICBEN and Inter-Noise were not additionally searched. Papers in all languages were sought. See Web Appendix A for the complete list of search terms included. 

Five existing systematic reviews of the evidence specifically regarding wind turbine noise effects on quality of life, wellbeing, mental health were identified [11,12,13,14,15]. Therefore, for wind turbine noise a review of existing systematic reviews rather than primary research papers was undertaken. 

For other environmental noise sources (road traffic, aircraft, railway) we sought to identify original research papers of quantitative design, on the effect of environmental noise on quality of life, wellbeing and mental health outcomes. Initial searches for systematic reviews in the field of environmental noise effects on mental health identified only one existing systematic review which drew conclusions about the strength of the evidence for environmental noise effects on child and adult mental health [16]. Therefore, a new search for primary papers was conducted from January 2005 onwards to build on the existing systematic review of the field. Papers up to start of October 2015 were included in the review. The reference lists of identified papers were also checked for further relevant citations. Grey-literature was not sought to be included in the review; an exception was made for the NORAH study as the Guideline Development Group felt this to be an important high profile study which was reporting at the time the review was concluded.

The methodology for the review, covering the review process, data extraction, and the evaluation of the quality of the evidence has already been described in the sister paper [1], along with the GRADE methodology [17] and its application in the review process. 

## 3. Results

### 3.1. Papers Identified

In total, 728 citations were identified from a search of the databases: after removal of 13 duplicates this left 715 citations identified from the database search. Following this systematic process of searching for papers, six additional papers were added after data extraction of the papers identified by the database search. The first was Belojevic et al., 2012 [17] which was co-authored by one of the reviewers and confirmed as relevant to our review: this paper was missed as the paper title refers to ‘executive functioning’ but also includes mental health and wellbeing outcomes. The second and third were Hjortebjerg et al., 2015 [18] and Roswall et al., 2015 [19] which were published online in mid-2015 after the search had been completed. The fourth was identified by a GDG panel member who reviewed a draft of the report [20]: it is not clear why this paper was not identified in the original search. The fifth was a report from the NORAH project, reporting on quality of life outcomes, which was published late in 2015 after the search had been completed [21]. The sixth was a paper published in 2014 [22]; and was not picked up in the search carried out at the end of the review process in October 2015. 

**Figure 1:**
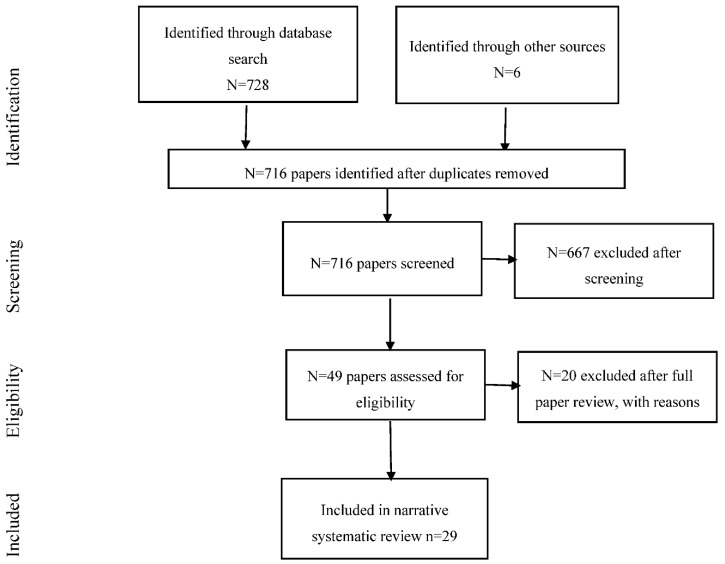
Flow chart showing the review process for the quality of life, wellbeing, and mental health papers.

Screening of the citations identified 49 that were potentially eligible: 29 were included and 20 were excluded after full text retrieval. Reasons for exclusions included that the study did not measure noise exposure, quality of life, wellbeing or mental health, that it was a review paper, or an experimental study. This led to a total of 29 primary research papers for inclusion in the review (see Figure 1). Web Appendix B presents the risk of bias assessment for each of these individual papers. 

### 3.2. Summary of Papers

Table 1 describes the papers identified. Nearly all the studies were cross-sectional (90%); there were more longitudinal studies (21%) than intervention studies (3%). Approximately, two-thirds (69%) of the papers were of adult populations, with one-third (34%) of child populations. 

Most studies examined road traffic noise exposure (83%) or aircraft noise exposure (41%). Nearly two-thirds of the papers used a L_Aeq_ noise metric (62%). Only 5 studies examined railway noise and 3 studies examined co-exposure with air pollution. Most papers focused on the home environment (97%), but one-third considered school exposure for children (34%).

A range of quality of life, wellbeing and mental health outcomes had been examined. The most commonly reported were self-reported quality of life (wellbeing, health status, vitality) using validated well-established scales (59%) such as the Short Form Health Survey (SF-36) and the General Health Questionnaire: however, the scales employed across these studies varied, making comparison across studies challenging. Eight papers (28%) considered emotional and conduct disorders in children usually measured with the Strengths and Difficulties questionnaire: a further five papers (17%) examined hyperactivity symptoms in children. Outcomes more indicative of psychiatric health were less reported, with 10% of papers reporting on medication use, 14% reporting self-report of anxiety or depression symptoms, and 7% reporting interview measures of depressive and anxiety disorders. 

The evaluation concluded that the majority of the studies were adequate in terms of taking sociodemographic confounding between noise exposure and mental health/wellbeing into account. 

### 3.3. Evaluating the Quality of the Evidence

The following sections summarize the quality of the evidence for environmental noise effects on self-reported quality of life or health; medication intake for treatment of anxiety and depression; self-reported depression, anxiety and psychological symptoms; interview measures of depressive and anxiety disorders; emotional and conduct disorders in children; hyperactivity in children. 

The GRADE methodology used to assess the quality of the evidence is described in the sister paper [17]. An overview of the ratings for the quality of the evidence for the different quality of life, wellbeing and mental health domains is given in Table 2. 

### 3.4. Findings of the Previous Systematic Review

This systematic review searched for papers published since 2005, as an existing systematic review had already identified papers examining environmental noise exposure effects on mental health published up to 2005 [16]. This previous systematic review identified 11 papers that examine the effects of chronic noise exposure on mental health of children or adults, as well as one narrative review. Only two studies identified in this previous systematic review were of longitudinal design; the rest of the studies were cross-sectional studies. The main findings of the review were that there was some supporting evidence of an effect of environmental noise on mental health: however, the evidence was less consistent for children than for adults. 

The previous systematic review did not differentiate the range of psychological outcomes being considered in the current review, which limits our ability to draw the conclusions of the two reviews together. However, where possible, we will contrast the conclusions of the two reviews. 

### 3.5. Self-Reported Quality of Life or Health

We identified 17 studies of associations of environmental noise on self-reported quality of life: 14 studies were of adult populations [19,20,21,22,23,24,25,26,27,28,29,30,31,32]: and three studies were of child populations [33,34,35]. The studies were predominantly cross-sectional: only one longitudinal study of an intervention, and three longitudinal prospective cohort studies were identified (however, it should be noted that the Schreckenberg et al., 2015 study is a complex design of repeated cross-sectional surveys, which contain a sub-sample of individuals being followed across waves). The detailed data extraction for each of these studies is given in Supplementary Table S1, organised by noise source, population (child or adult), and study design. 

The GRADE evaluation of these papers is given in Table 3. The risk of bias was judged to be high in these individual studies. The evidence was predominantly from studies of cross-sectional design, with only one intervention and two longitudinal studies; many of the studies report poor response rates (<40%) which may lead to bias or fail to report any response rate information; some studies are of very small samples. Overall, noise exposure assessment was based upon long-term measurement data using established metrics, airport contour data, or good quality noise modeling. Most, but not all studies made good adjustment for socioeconomic and other confounders.

#### 3.5.1. Aircraft Noise Exposure

There were seven studies that examined the associations of aircraft noise exposure with self-reported quality of life or health, of which three were of child populations—all reporting on the RANCH project. See Supplementary Table S1 for detailed data extraction for these papers. Of these seven studies, five studies found no association between aircraft noise exposure and poorer quality of life or self-rated health and two studies demonstrated an association. 

Applying the GRADE framework to assess the quality of evidence across the available studies of aircraft noise on self-reported quality of life or health (Table 3), we considered longitudinal or intervention studies the ideal study design. However, as only cross-sectional studies were available, which we designated as low quality. We downgraded the evidence to very low quality evidence given issues with inconsistency in findings across studies and the high risk of bias. We concluded that there is very low evidence for no substantial effect of aircraft on self-reported quality of life or health.

#### 3.5.2. Road Traffic Noise Exposure

We identified 14 studies examined associations between road traffic noise exposure and self-rated quality of life or health. Of these studies, nine suggest no significant association between road traffic noise exposure and self-rated quality or life or health, including the only intervention study and one longitudinal prospective study; four studies suggest a significant association, although often only in particular sub-samples, such as males or noise sensitive individuals. 

For the quality of evidence across the available studies of road traffic noise associated with self-rated quality of life or health, adapting the GRADE approach, we considered longitudinal or intervention studies the ideal study design and designation this evidence as high quality (Table 3). However, this rating was downgraded to low quality given issues with inconsistency in findings across the studies and high risk of bias. No reasons to upgrade the evidence were identified. We concluded that there is very low quality evidence for no substantial effect of road traffic noise exposure on self-reported quality of life or health. 

#### 3.5.3. Railway Noise Exposure

Three studies of railway noise exposure and self-rated quality of life or health were identified, of which only one was longitudinal. Two of the studies found evidence for an association of railway noise on self-rated quality of life or health. 

For the quality of evidence across the available studies of railway noise effects on quality of life or self-reported health, adapting the GRADE approach, we considered longitudinal studies the ideal study design and designated evidence from the longitudinal study as high quality (Table 3). However, this rating was downgraded to low quality given issues with inconsistency of findings across the studies and high risk of bias. There was no reason for upgrading. We concluded that there is very low quality evidence for an effect of railway noise exposure on self-reported quality of life or health, albeit from a limited number of studies.

### 3.6. Medication Intake for Treatment of Anxiety and Depression 

We identified three studies of noise effects on medication intake for treatment of anxiety and depression [25,36,37]. The studies were all of European adult populations. All the studies were cross-sectional. The studies examine medication intake for anxiolytics/hypnotics (typically used to treat anxiety problems) and psychotropic medication use, covering a range of drug treatments for depression and anxiety, as well as other psychiatric disorders. One study was of aircraft noise exposure and three examined road traffic noise exposure, with one study focusing specifically on night-time road traffic noise exposure. No studies of railway noise exposure were identified. See Supplementary Table S2 for the detailed data extraction for each of these papers. 

The risk of bias in these individual studies was judged to be low, with studies having good noise exposure assessment, making adjustment for socioeoconomic and other confounders, and making use of medication registers in most studies. 

#### 3.6.1. Aircraft Noise Exposure

We identified one cross-sectional study of aircraft noise exposure and use of prescription medication, which found no association of aircraft noise exposure on medication use for anxiety and depression. No evidence from longitudinal or intervention studies was available. 

However, as only one observational cross-sectional study the evidence was designated as low quality evidence (Table 4). This was downgraded to very low quality as we are unable to evaluate consistency of results across studies. The review concludes that the evidence is low quality for an effect of aircraft noise on medication intake for depression and anxiety. 

#### 3.6.2. Road Traffic Noise Exposure

We identified three cross-sectional studies of road traffic noise exposure and medication use for depression and anxiety. Of these, two studies found no association between road traffic noise exposure and self-reported medication use for anxiety or depression: one study found an association but only for a specific sub-sample: those from lower social position. 

As there were only cross-sectional studies, the evidence was evaluated as low quality evidence. Given the inconsistent findings across the studies, this was further downgraded, concluding that there is very low quality evidence for no substantial effect of road traffic noise on medication intake for depression and anxiety. 

#### 3.6.3. Railway Noise Exposure

No studies of railway noise exposure and medication intake for depression and anxiety were identified, so no GRADE evaluation on the quality of the evidence available was possible.

### 3.7. Self-Reported Depression, Anxiety and Psychological Symptoms

We identified four studies of associations between environmental noise exposure and self-reported depression, anxiety and psychological symptoms [26,27,32,38]. All the studies were cross-sectional, with the exception of one intervention study. These studies used established self-report measures of depression, anxiety and psychological symptoms such as the General Health Questionnaire or the Hopkins Symptom Checklist-25. Supplementary Table S3 shows the data extraction for these studies. The studies only examined road traffic noise. The GRADE evaluation of these papers is given in Table 5.

The risk of bias was judged to be high for these individual studies. The evidence was predominantly cross-sectional, and one study poorly reports the noise metrics or modeling undertaken: nor does this study differentiate air pollution from noise exposure. Further, whilst participants are usually identified by the random selection of homes within a geographical area, some of the response rates for the studies are low. The studies made good adjustment for socioeconomic confounding and used established assessments of depression, anxiety and psychological symptoms. 

#### 3.7.1. Aircraft Noise Exposure

No studies of aircraft noise exposure and self-reported depression, anxiety and psychological symptoms were identified, so no GRADE evaluation of the quality of the evidence across studies was possible.

#### 3.7.2. Road Traffic Noise Exposure

We identified one intervention and three cross-sectional studies reporting on associations between road traffic noise exposure and self-reported depression, anxiety and psychological symptoms. Of these, two out of the four studies, including the intervention study, found no association between road traffic noise exposure and self-reported depression, anxiety and psychological symptoms; two of these four studies found an association or trend but only for specific sub-samples of the population such as those with noise sensitivity or poor sleep quality. 

The quality of the evidence for road traffic noise effects on self-reported depression, anxiety and psychological symptoms, was evaluated as high quality (Table 5). Given the high risk of bias, inconsistency across studies, and precision, the final evaluation concluded that there is very low quality evidence that there is no substantial effect of road traffic noise exposure on depression, anxiety, and psychological distress.

#### 3.7.3. Railway Noise Exposure

No studies of railway noise exposure and self-reported depression, anxiety and psychological symptoms were identified, so no GRADE evaluation on the quality of the evidence available was possible.

### 3.8. Interview Measures of Depression and Anxiety

We identified two studies that examined the association of environmental noise exposure on interview assessments of depression and anxiety disorders (often referred to as ‘common mental disorders’ in the literature) [32,39]. Both studies were of adult populations: one study was an intervention study and the other was a cross-sectional study. One study examined road traffic noise and the other aircraft noise. Supplementary Table S4 gives the detailed data extraction for these studies. 

The risk of bias was judged to be high for these individual studies. It was not clear in one of the studies how noise exposure had been assessed and it was also felt that this study would benefit from further adjustment for socioeconomic factors. In the studies, whilst participants were usually identified by the random selection of homes within a geographical area, with good response rates, the samples were very small. 

#### 3.8.1. Aircraft Noise Exposure

We identified one cross-sectional study which examined aircraft noise exposure and associations with interviewer assessed depression and anxiety disorders: this study supported the hypothesis that noise is associated with depression and anxiety disorders. However, these conclusions may be biased by the small sample and noise exposure assessment.

For the quality of the evidence across the available studies for aircraft noise effects on interview measures of depression or anxiety, adapting the GRADE approach, we considered longitudinal studies the ideal study design and would designate evidence from longitudinal studies as high quality (Table 6). As we only had cross-sectional evidence, this was rated as low quality, and was further downgraded to very low quality based on the high risk of bias, being unable to assess consistency across studies, and precision in the study. We found no reasons to upgrade the evidence. We concluded that there is very low quality evidence for an effect of aircraft noise exposure on interview measures of depression and anxiety.

#### 3.8.2. Road Traffic Noise Exposure

We identified one intervention study that examined road traffic noise exposure and associations with interviewer assessed depression and anxiety disorders: this study did not find evidence for an association. 

Initially, the evidence was evaluated as high quality (Table 6). This was downgraded to very low quality based on some risk of bias, being unable to assess consistency across studies, and precision in the study. We concluded that there is very low quality evidence that there is no substantial effect of road traffic noise exposure on interview measures of depression and anxiety.

#### 3.8.3. Railway Noise Exposure

No studies of railway noise exposure and interview measures of depression and anxiety were identified, so no GRADE evaluation of the quality of the evidence was possible.

### 3.9. Emotional and Conduct Disorders in Children 

We identified eight studies that examined the association of environmental noise exposure on emotional and conduct disorders in children [18,33,35,40,41,42,43,44]. These studies mainly use the Strengths and Difficulties Questionniare (SDQ), which gives a total score of psychological distress, as well as scores on 5 sub-scales: conduct problems, emotional symptoms, peer-problems, prosocial behavior and hyperactivity. Hyperactivity was considered as an outcome in its own right (see Section 3.9). Most of the studies were cross-sectional. None of the studies was of an intervention and only two studies were of a longitudinal design. Five studies examined aircraft noise exposure (albeit it, all examining the RANCH data), seven studies examined road traffic noise exposure and one study examined railway noise exposure. All of the studies were of European populations. Supplementary Table S5 gives the detailed data extraction for these studies. 

The risk of bias in these individual studies was judged to be low. These studies had good noise characterization based on long-term measurement or modeling, with adjustment for socioeconomic confounding. Participants were usually identified by the random selection of schools or homes within a geographical area, with good response rates. Established, age-appropriate tests of psychological health have been employed. However, many studies use parent or teacher assessments of psychological health, which may be biased, but this is an established approach for assessing child psychological health. 

#### 3.9.1. Aircraft Noise Exposure

We identified five studies that examined aircraft noise exposure, with these studies all reporting analyses of the RANCH data. One of these studies was longitudinal. None of these studies found an association between aircraft noise exposure and psychological distress as assessed by the total score of the SDQ.

For the quality of evidence available across the studies for aircraft noise effects on emotional and conduct disorders in children, adapting the GRADE approach, we considered longitudinal studies the ideal study design and designate evidence from longitudinal studies as high quality (Table 7). We further downgraded for being unable to assess inconsistency (all the papers were of the same data) and precision, and therefore the final rating was low quality. We concluded that there is low quality evidence that there is no substantial effect of aircraft noise on emotional and conduct disorders in childhood. 

#### 3.9.2. Road Traffic Noise Exposure

Seven studies examined road traffic noise. Six studies report a significant association between road traffic noise, including the only longitudinal study but some studies do not find an association for all the aspects of children’s psychological health examined. 

For the quality of the evidence available across studies of road traffic noise effects on emotional and conduct disorders in children, adapting the GRADE approach, we considered longitudinal studies the ideal study design and designated evidence from longitudinal studies as high quality (Table 7). It was necessary to downgrade the evidence to moderate quality based on the inconsistent findings across the studies available. We conclude that there is moderate quality evidence for an effect of road traffic noise on emotional and conduct disorders in childhood. 

#### 3.9.3. Railway Noise Exposure

We identified one longitudinal study examined railway noise exposure, which suggested some significant associations. 

For the quality of the evidence available for railway noise effects on emotional and conduct disorders in children, adapting the GRADE approach, we considered longitudinal studies the ideal study design and designated evidence from longitudinal studies as high quality (Table 7). We downgraded this to moderate quality evidence because we were unable to assess inconsistency and heterogeneity of findings across studies, as only one study was available. We concluded that there is moderate quality evidence for an effect of railway noise on emotional and conduct disorders in childhood. 

### 3.10. Hyperactivity in Children 

We identified five studies that examined the association of environmental noise exposure on hyperactivity in children [18,40,41,42,43]. These studies all use the hyperactivity sub-scale of the Strengths and Difficulties Questionniare (SDQ). Four of the studies reported cross-sectional associations and two papers reported longitudinal associations. No studies were of interventions. All of the studies were of European populations. Three studies were of aircraft noise exposure, with these studies all reporting analyses of the RANCH data and five studies that examined road traffic noise. One study of railway noise exposure was identified. Supplementary Table S6 gives the detailed data extraction for these studies. Methodologically, these studies were evaluated as robust (good noise characterization, low risk of bias, adjustment for confounding, random selection, good response rates). 

#### 3.10.1. Aircraft Noise Exposure

The three studies examining aircraft noise exposure all reported analyses of the RANCH data. Two studies report the same significant cross-sectional association, and one study reported no significant longitudinal association.

For the quality of the evidence across the available studies for aircraft noise effects on hyperactivity in children, adapting the GRADE approach, we considered longitudinal studies the ideal study design and designated evidence from longitudinal studies as high quality (Table 8). It was necessary to downgrade the evidence to low quality based on the GRADE consistency and precision criteria across the studies available. We concluded that there is low quality evidence that there is an effect of aircraft noise on hyperactivity symptoms in children. 

#### 3.10.2. Road Traffic Noise Exposure

We identified one longitudinal and three cross-sectional studies that examined road traffic noise. Of these, two studies report no significant association, albeit on the same data and two papers report a significant association. 

For the quality of the evidence available across the studies available for road traffic noise effects on hyperactivity in children, adapting the GRADE approach, we considered longitudinal studies the ideal study design and designated evidence from longitudinal studies as high quality (Table 8). It was necessary to downgrade the evidence to moderate quality based on the inconsistency of results across studies. We concluded that there is moderate quality evidence that there is an effect of road traffic noise on hyperactivity symptoms in children.

#### 3.10.3. Railway Noise Exposure

The one longitudinal study identified that examined railway noise exposure and hyperactivity, suggested no significant association. 

For the quality of the evidence for railway noise effects on hyperactivity in children, adapting the GRADE approach, we considered longitudinal studies the ideal study design and designated evidence from longitudinal studies as high quality (Table 8). We further downgraded this to moderate quality, as we cannot assess consistency across study findings, as there is only one study. We concluded that there is moderate quality evidence that there is no substantial effect of environmental noise exposure from railway noise on hyperactivity symptoms in children.

### 3.11. Review of Evidence from Systematic Reviews of Wind Turbine Noise on Quality of Life, Wellbeing and Mental Health

We identified five existing systematic reviews that examined wind turbine noise effects on adult mental health and wellbeing, and were considered of sufficient quality according to the AMSTAR tool [11,12,13,14,15]. There is inconsistent evidence from systematic reviews that wind turbine noise exposure is associated with poorer quality of life, wellbeing and mental health. 

There is consensus across the systematic reviews that examine the influence of wind turbine noise on mental health and wellbeing that there are a limited number of peer-reviewed studies available and that those available are not methodologically robust: many published studies are poor quality cohort and case-control studies. Only two of the systematic reviews examined the quality of the available studies.

We also rated the risk of bias in the individual papers included in these systematic reviews as being high. Many of the studies described in the reviews use distance from a wind farm to determine audible noise exposure with only a few more recent studies estimating noise exposure at the respondents’ residences. Estimating exposure is an essential part of the evidence chain, if recommendations regarding limit values are to be determined. Studies also tend to make poor adjustment for socioeconomic and other important confounders such as existing health and noise annoyance. Some studies have poor response rates, which can lead to bias. Most studies are small scale, where the exposed and control populations are not well defined, which can lead to bias and some do not report any response rate information. Some studies are of very small samples. The systematic reviews identify information and response bias as particular issues. While some studies use established measures of health status (e.g., SF-36, GHQ) or quality of life, other studies use non-validated questions about individual symptoms. 

None of the systematic reviews reported effect sizes across studies using meta-analysis techniques: this is due to the low number of studies available on which to base effect estimates in some reviews, as well as differences in methodologies between studies. The lack of meta-analyses means that we do not have an estimate of the exposure-response relationships for wind turbine noise effects on various quality of life, wellbeing and mental health, outcomes. There is no consistent high quality evidence that with every unit increase of wind turbine noise, psychological distress increases or mental health decreases or quality of life decreases. 

The evidence should be considered incomplete: further robust epidemiological cohort studies of exposed populations, including children, adults, the elderly, and vulnerable populations are required: those with pre-existing mental or physical health issues should be examined. Future studies need to account for a range of potential confounding factors, including noise annoyance, as well as sociodemographic factors, and visual factors associated with wind turbines. Evidence is also needed that addresses a wide range of assessments of mental health and wellbeing. Current evidence tends to use either individual self-report questions or scales assessing symptom reports relating to quality of life, mental health or psychological distress. Studies need to address clinically significant, objectively rated assessments of mental health and psychological distress such as ICD-10/DSM-V diagnoses of depression and anxiety, as well as medication use for mental health issues. No studies to date have assessed clinical mental health outcomes such as ICD-10 psychiatric diagnoses. Few studies relating to the health effects of infrasound or low frequency noise associated with wind turbines were identified: further research should also focus on this area, as well as examining the mechanisms by which infrasound or low frequency noise might influence human health. 

According to GRADE, longitudinal studies would yield high quality evidence; we only identified observational studies, which yield at most low quality evidence unless we can upgrade them (Table 9). We downgraded the evidence further and rated the quality of the evidence as very low quality. This decision was based upon study limitations, inconsistency and indirect comparisons across studies. We concluded that there is very low quality evidence for no substantial effect of wind turbine noise on quality of life, wellbeing or mental health. 

The available evidence relating to wind turbine noise effects on mental health and wellbeing is not sufficient to warrant the formulation of recommendations of guidelines for community noise exposure for this source. 

## 4. Discussion

Following use of the GRADE methodology, the systematic review draws the following conclusions to feed into the revision of the WHO Guidelines.

- There was very low quality evidence across the available studies for no substantial effect of aircraft noise or road traffic noise on poorer quality of life or health. There was very low quality evidence across the available studies for an effect of railway noise on poorer quality of life or health.
- There was very low quality evidence across the available studies for an effect of aircraft noise on medication intake for depression and anxiety. There was very low quality evidence across the available studies for no substantial effect of road traffic noise on medication intake for depression and anxiety. No studies of railway noise on medication intake were identified.
- There was very low quality evidence across the available studies for no substantial effect of road traffic noise on self-reported depression or anxiety. No studies of aircraft noise or railway noise on self-reported depression or anxiety were identified.
- There was very low quality evidence across the available studies for no substantial effect of road traffic on interview measures of depression or anxiety. There was very low quality evidence across the available studies for an effect of aircraft noise on interview measures of depression or anxiety. No studies of railway noise on interview measures of depression or anxiety were identified.
- There was moderate quality evidence across the available studies for an effect of road traffic and railway noise on emotional and conduct disorders in children; and low quality evidence across the available studies for no substantial effect of aircraft noise on emotional and conduct disorders in children.
- There was low quality evidence across the available studies for an association of aircraft noise and moderate quality evidence for an association of road traffic noise on hyperactivity in children. There was moderate quality evidence across the available studies for no substantial association of railway noise on hyperactivity in children.
- There was very low quality evidence, drawn from existing systematic reviews, for no substantial effect of wind turbine noise on quality of life, wellbeing or mental health.

As previously described, several pathways and mechanisms for the effects of noise on environmental noise on quality of life, wellbeing and mental health have been put forward [6,7]. Noise, as an environmental stressor, could lead to an increase in stress hormones and cortisol, leading to the development of depression and anxiety disorders. Psychological stress responses, such as annoyance, may also directly activate physiological stress hormones. However, the evidence for an association of environmental noise exposure and elevated levels of these stress hormones is mixed [8]. Whether an individual experiences stress responses when exposed to chronic environmental noise depends on a myriad of other factors including prior history of mental ill-health; physical ill-health; appraisal of the noise (e.g., fear, meaning, control); and coping strategies. There may also be selection out of noisy areas for those who can’t cope and poor mental health may exacerbate other health effects of noise e.g., on annoyance and sleep effects. Night-time noise might interfere with sleep, which can cause low mood and fatigue the next day and may particularly impact those with existing ill-health. Children in particular are often thought to be more vulnerable to the effects of environmental noise because of less well-developed coping strategies. 

Key limitations of the available evidence include a lack of studies per se: many studies are also limited by small sample sizes. As described in the previous review of this field [16] and the sister systematic review paper [1], there is a lack of intervention studies, longitudinal studies, exposure-response relationships for quality of life, wellbeing and mental health outcomes. A further limitation is the use of the GRADE methodology, designed to evaluate clinical practice recommendations: it has been adapted here to evaluate epidemiological evidence, which may or may not be appropriate. 

A major limitation to this systematic review is the lack of studies in many of the domains examined. It is challenging to draw conclusions about the quality and strength of the evidence: there are currently too few studies available in many of the domains, when considered by specific noise source and outcome as required by GRADE. The conclusions of the current review, broadly agree and build on those of the previous systematic review [16], which concluded that there was some evidence for effects of environmental noise on child and adult mental health but that overall the evidence was equivocal. A recent systematic review published whilst this review was ongoing which focused only on studies of noise and children’s mental also reaches a similar conclusion [45]. The current review suggests that evidence for some areas has strengthened in the past decade, e.g., road traffic noise and children’s emotional and conduct disorders, and hyperactivity; aircraft noise and medication use for depression and anxiety; aircraft noise and interview measures of depression and anxiety. However, the current review also highlights the paucity of evidence for some noise sources, particularly railway noise exposure and for some mental health outcomes. 

The review does not take into account evidence published before January 2005 or after October 2015. It is worth noting that several good quality studies of environmental noise have been published since this systematic review was conducted, including additional longitudinal studies and studies of co-exposure, that might have added to the evidence base for some sources and outcomes, for example, but not limited to [46,47,48]. 

The field is not yet at a stage where meta-analyses could be conducted. This is because studies use a wide-range of outcome measures and also differ in how they assess or characterise noise. In terms of mental health, the advantages of using an established scale to assess children’s mental health, the Strengths and Difficulties Questionnaire, is starting to be seen, as we can more easily compare findings across studies. There are a large number of standardized tests for adult mental health outcomes currently available: some of which assess symptoms some which diagnoses. It may be worth researchers in the field debating which assessments to include in their studies, to enable comparison between studies. Yet other differences in study design mean that it is still challenging to perform meta-analyses. These issue is discussed further in the sister paper [1]. 

The papers identified almost all focus on using noise metrics based on average sound pressure levels over a given period of time, such as the day-time or night-time period. Other noise metrics need to be explored in relation to quality of life and mental health outcomes. This may be particularly relevant for studies of wind-turbine noise exposure, where the sound level of the exposure is moderate. Methodologically, studying the impact wind-turbine noise is uniquely challenging, as it is difficult to obtain data from before the installation or announcement of the intention to install wind-turbines. 

Many of the studies of environmental noise effects on quality of life, wellbeing and mental health do not take into account an individual’s history of mental ill-health, their ability to cope, their annoyance responses or their appraisal of the noise. These may be important confounding factors in the association and current studies may be over-simplifying the relationship between environmental noise and mental health. 

## 5. Conclusions

In terms of environmental noise effects on quality of life, wellbeing and mental health, this review has found that the quality of the evidence when considered across the studies is of moderate quality for a couple of outcomes, e.g., road traffic noise effects on emotional and conduct disorders in children and hyperactivity in children, but is of weaker quality, indicative of effects or no substantial effects for other outcomes and noise sources. These conclusions, regarding the quality of the evidence, are limited by the low number of studies for many of the outcomes. Overall, environmental noise effects on quality of life, wellbeing, and mental health is a field of research characterized by a lack of longitudinal and intervention studies: there are also only a small number of studies of clinically significant mental health outcomes; few studies of railway noise exposure; and studies of larger, representative samples are needed. The lack of evidence across studies for noise effects for many of the quality of life, wellbeing, and mental health domains examined does not necessarily mean that there are no effects: rather, that they have not yet been studied robustly. 

## Figures and Tables

**Table 1:** Summary of key features of studies of quality of life, wellbeing and mental health.

	Number of Papers Out of 29	% of 29 Papers *
**NOISE EXPOSURE**		
Road noise	24	83
Aircraft noise	12	41
Rail noise	5	17
Co-exposures, e.g., air pollution	3	10
**STUDY DESIGN**		
Cross-sectional	26	90
Longitudinal	6	21
Intervention	1	3
**NOISE METRIC**		
L_Aeq_	18	62
L_dn_/L_den_	13	45
L_night_	7	24
**SETTING**		
School	8	28
Home	28	97
**POPULATION**		
Adults	20	69
Children	10	34
**OUTCOME**		
Self-reported quality of life (well-being, health status, vitality) using assessments such as the Short Form Health Survey (SF-36), General Health Questionnaire (GHQ), WHO Quality of Life assessment (WHOQOL and WHOQOL-BREF), Health-related Quality of Life (HRQOL)	17	59
Medication intake for treatment of anxiety and depression	3	10
Self-reported depression, anxiety and psychological symptoms (scale)	4	14
Interview measures of depressive and anxiety disorders	2	7
Emotional and conduct disorders in children (e.g., assessed by instruments such as strengths and difficulties questionnaire and KINDL)	8	28
Hyperactivity (assessed by validated scale)	5	17

* total % within categories, e.g., POPULATION will not add to 100% as some studies fall within more than one category.

**Table 2:** Summary of quality of the evidence and assessment of effect for environmental noise effects on quality of life, wellbeing and mental health.

	Environmental Noise Exposure		
**Outcome Domain**	**Aircraft Noise** **Quality of Evidence & Assessment of Effect**	**Road Traffic Noise** **Quality of Evidence & Assessment of Effect**	**Railway Noise** **Quality of Evidence & Assessment of Effect**
**Self-reported quality of life or health**	Very low quality–no effect	Low quality–no effect	Low quality–harmful effect
**Medication intake for treatment of anxiety and depression**	Very low quality–harmful effect	Very low quality–no effect	n.a.
**Self-reported depression, anxiety and psychological symptoms**	n.a.	Very low quality–no effect	n.a.
**Interview measures of depressive and anxiety disorders**	Very low quality–harmful effect	Very low quality–no effect	n.a.
**Emotional conduct disorders in children**	Low quality–no effect	Moderate quality–effect	Moderate quality–harmful effect
**Hyperactivity**	Low quality–harmful effect	Moderate quality–harmful effect	Moderate quality–no effect

n.a. no studies available to evaluate.

**Table 3:** GRADE for the quality of evidence of environmental noise being associated with self-reported health and quality of life.

	AIRCRAFT NOISE (7 STUDIES)			ROAD TRAFFIC NOISE (13 STUDIES)			RAILWAY NOISE (3 STUDIES)		
Domains	Criterion	Assessment	Downgrading	Criterion	Assessment	Downgrading	Criterion	Assessment	Downgrading
Start Level	Intervention/Longitudinal	All Studies cross-Sectional	Low Quality	Intervention/Longitudinal	1 Intervention and 1 Longitudinal Study	High Quality	Intervention/Longitudinal	1 Longitudinal Study	High Quality
1. Study Design	Study quality & bias	High risk of bias	Downgrade	Study quality & bias	High risk of bias	Downgrade	Study quality & bias	High risk of bias	Downgrade
2. Inconsistency	Conflicting results; high I^2^	Inconsistent evidence; I^2^ not assessed	Downgrade	Conflicting results; high I^2^	Inconsistent evidence; I^2^ not assessed	Downgrade	Conflicting results; high I^2^	Inconsistent evidence; I^2^ not assessed	Downgrade
3. Indirectness	Direct comparison; same PECCO	No indirect comparisons made	No downgrade	Direct comparison; same PECCO	No indirect comparisons made	No downgrade	Direct comparison; same PECCO	Indirect comparisons made	No downgrade
4. Precision	Confidence interval contains 25% harm or benefit	Unable to rate for narrative review	No downgrade	Confidence interval contains 25% harm or benefit	Unable to rate for narrative review	No downgrade	Confidence interval contains 25% harm or benefit	Unable to rate for narrative review	No downgrade
5. Publication Bias	Funnel plot indicates	Suspected but unable to rate for narrative review	No downgrade	Funnel plot indicates	Suspected but unable to rate for narrative review	No downgrade	Funnel plot indicates	Suspected but unable to rate for narrative review	No downgrade
**Overall Judgement**			**Very Low Quality**			**Very Low Quality**			**Very Low Quality**
6. Dose-response	Significant trend	No	No upgrade	Significant trend	No	No upgrade	Significant trend	No	No upgrade
7. Magnitude of effect	RR > 2		No upgrade	RR > 2		No upgrade	RR > 2		No upgrade
8.Confounding adjusted	Effect in spite of confounding working towards the nil	Good control for confounding	No upgrade	Effect in spite of confounding working towards the nil	Good control for confounding	No upgrade	Effect in spite of confounding working towards the nil	Good control for confounding	No upgrade
**Overall Judgement**			**Very Low Quality**			**Very Low Quality**			**Very Low Quality**

**Table 4:** GRADE for the quality of evidence of environmental noise associated with medication intake for treatment of anxiety and depression.

	AIRCRAFT NOISE (1 STUDY)			ROAD TRAFFIC NOISE (3 STUDIES)		
Domains	Criterion	Assessment	Downgrading	Criterion	Assessment	Downgrading
Start Level	Longitudinal/Intervention	Cross-Sectional	Low Quality	Longitudinal/Intervention	Cross-Sectional	Low Quality
1. Study Design	Study quality & bias	Low risk of bias	No downgrade	Study quality & bias	Low risk of bias	No downgrade
2. Inconsistency	Conflicting results; high I^2^	I^2^ not assessed	Downgrade	Conflicting results; high I^2^	Inconsistent evidence; I^2^ not assessed	Downgrade
3. Indirectness	Direct comparison; same PECCO	Did not make indirect comparison	No downgrade	Direct comparison; same PECCO	Did not make indirect comparison	No downgrade
4. Precision	Confidence interval contains 25% harm or benefit	Unable to rate for narrative review	No downgrade	Confidence interval contains 25% harm or benefit	Unable to rate for narrative review	No downgrade
5. Publication Bias	Funnel plot indicates	Suspected but unable to rate for narrative review	No downgrade	Funnel plot indicates	Suspected but unable to rate for narrative review	No downgrade
**Overall Judgement**			**Very Low Quality**			**Very Low Quality**
6. Dose-response	Significant trend	Examined but only in small number of studies	No upgrade	Significant trend	Examined but only in small number of studies	No upgrade
7. Magnitude of effect	RR > 2	Unable to assess	No upgrade	RR > 2	Unable to assess	No upgrade
8. Confounding adjusted	Effect in spite of confounding working towards the nil	Good control for confounding	No upgrade	Effect in spite of confounding working towards the nil	Good control for confounding	No upgrade
**Overall Judgement**			**Very Low Quality**			**Very Low Quality**

**Table 5:** GRADE for the quality of evidence of environmental noise being associated with self-reported depression, anxiety and psychological symptoms.

	ROAD TRAFFIC NOISE (4 STUDIES)		
Domains	Criterion	Assessment	Downgrading
Start Level	Longitudinal/Intervention	1 Intervention Study	High Quality
1. Study Design	Study quality & bias	High risk of bias	Downgrade
2. Inconsistency	Conflicting results; high I^2^	Inconsistent evidence; I^2^ not assessed	Downgrade
3. Indirectness	Direct comparison; same PECCO	No indirect comparisons made	No downgrade
4. Precision	Confidence interval contains 25% harm or benefit	Serious	Downgrade
5. Publication Bias	Funnel plot indicates	Suspected but unable to rate for narrative review	No downgrade
**Overall Judgement**			**Very Low Quality**
6. Dose-response	Significant trend	Not assessed	No upgrade
7. Magnitude of effect	RR > 2	Not assessed	No upgrade
8. Confounding adjusted	Effect in spite of confounding working towards the nil	Adjusted	No upgrade
**Overall Judgement**			**Very Low Quality**

**Table 6:** GRADE for the quality of evidence of environmental noise being associated with interview measures of depression and anxiety.

	AIRCRAFT NOISE (1 STUDY)			ROAD TRAFFIC NOISE (1 STUDY)		
Domains	Criterion	Assessment	Downgrading	Criterion	Assessment	Downgrading
Start Level	Longitudinal/Intervention	Cross-Sectional	Low Quality	Longitudinal/Intervention	1 Longitudinal Study	High Quality
1. Study Design	Study quality & bias	High risk of bias	Downgrade	Study quality & bias	Some risk of bias	Downgrade
2. Inconsistency	Conflicting results; high I^2^	I^2^ not assessed	Downgrade	Conflicting results; high I^2^	Inconsistent evidence; I^2^ not assessed	Downgrade
3. Indirectness	Direct comparison; same PECCO	No indirect comparisons made	No downgrade	Direct comparison; same PECCO	No indirect comparisons made	No downgrade
4. Precision	Confidence interval contains 25% harm or benefit	Serious imprecision of results	Downgrade	Confidence interval contains 25% harm or benefit	Unable to rate for narrative review	Downgrade
5. Publication Bias	Funnel plot indicates	Suspected but unable to rate for narrative review	No downgrade	Funnel plot indicates	Suspected but unable to rate for narrative review	No downgrade
**Overall Judgement**			**Very Low Quality**			**Very Low Quality**
6. Dose-response	Significant trend	Not assessed	No upgrade	Significant trend	Not assessed	No upgrade
7. Magnitude of effect	RR > 2	No	No upgrade	RR > 2	No	No upgrade
8. Confounding adjusted	Effect in spite of confounding working towards the nil	Residual confounding may remain	No upgrade	Effect in spite of confounding working towards the nil	Good control for confounding	No upgrade
**Overall Judgement**			**Very Low Quality**			**Very Low Quality**

**Table 7:** GRADE for the quality of evidence of environmental noise being associated with emotional and conduct disorders in children.

	AIRCRAFT NOISE (5 STUDIES)			ROAD TRAFFIC NOISE (7 STUDIES)			RAILWAY NOISE (1 STUDY)		
Domains	Criterion	Assessment	Downgrading	Criterion	Assessment	Downgrading	Criterion	Assessment	Downgrading
Start Level	Longitudinal	1 Longitudinal Study	High Quality	Longitudinal	1 Longitudinal Study	High Quality	Longitudinal	1 Longitudinal Study	High Quality
1. Study Design	Study quality & bias	Low risk of bias	No downgrade	Study quality & bias	Low risk of bias	No downgrade	Study quality & bias	Low risk of bias	No downgrade
2. Inconsistency	Conflicting results; high I^2^	I^2^ not assessed	Downgrade	Conflicting results; high I^2^	Inconsistent evidence; I^2^ not assessed	Downgrade	Conflicting results; high I^2^	Inconsistent evidence; I^2^ not assessed	Downgrade
3. Indirectness	Direct comparison; same PECCO	No indirect comparisons made	No downgrade	Direct comparison; same PECCO	No indirect comparisons made	No downgrade	Direct comparison; same PECCO	No indirect comparisons made	No downgrade
4. Precision	Confidence interval contains 25% harm or benefit	Unable to rate for narrative review	Downgrade	Confidence interval contains 25% harm or benefit	Unable to rate for narrative review	No downgrade	Confidence interval contains 25% harm or benefit	Unable to rate for narrative review	No downgrade
5. Publication Bias	Funnel plot indicates	Suspected but unable to rate for narrative review	No downgrade	Funnel plot indicates	Suspected but unable to rate for narrative review	No downgrade	Funnel plot indicates	Suspected but unable to rate for narrative review	No downgrade
**Overall Judgement**			**Low Quality**			**Moderate Quality**			**Moderate Quality**
6. Dose-response	Significant trend	Limited evidence	No upgrade	Significant trend	Limited evidence	No upgrade	Significant trend	Limited evidence	No upgrade
7. Magnitude of effect	RR > 2	No	No upgrade	RR > 2	No	No upgrade	RR > 2	No	No upgrade
8. Confounding adjusted	Effect in spite of confounding working towards the nil	Good control for confounding	No upgrade	Effect in spite of confounding working towards the nil	Good control for confounding	No upgrade	Effect in spite of confounding working towards the nil	Good control for confounding	No upgrade
**Overall Judgement**			**Low Quality**			**Moderate Quality**			**Moderate Quality**

**Table 8:** GRADE for the quality of evidence of environmental noise being associated with hyperactivity in children.

	AIRCRAFT NOISE (3 STUDIES)			ROAD TRAFFIC NOISE (4 STUDIES)			RAILWAY NOISE (1 STUDY)		
Domains	Criterion	Assessment	Downgrading	Criterion	Assessment	Downgrading	Criterion	Assessment	Downgrading
Start Level	Longitudinal/Intervention	1 Study	High Quality	Longitudinal/Intervention	1 Study	High Quality	Longitudinal/Intervention	1 Study	High Quality
1. Study Design	Study quality & bias	Low risk of bias	No downgrade	Study quality & bias	Low risk of bias	No downgrade	Study quality & bias	Low risk of bias	No downgrade
2. Inconsistency	Conflicting results; high I^2^	Inconsistent evidence; I^2^ not assessed	Downgrade	Conflicting results; high I^2^	Inconsistent evidence; I^2^ not assessed	Downgrade	Conflicting results; high I^2^	I^2^ not assessed	Downgrade
3. Indirectness	Direct comparison; same PECCO	No indirect comparisons made	No downgrade	Direct comparison; same PECCO	No indirect comparisons made	No downgrade	Direct comparison; same PECCO	No indirect comparisons made	No downgrade
4. Precision	Confidence interval contains 25% harm or benefit	serious imprecision of results	Downgrade	Confidence interval contains 25% harm or benefit	Unable to rate for narrative review	No downgrade	Confidence interval contains 25% harm or benefit	Unable to rate for narrative review	No downgrade
5. Publication Bias	Funnel plot indicates	Suspected but unable to rate for narrative review	No downgrade	Funnel plot indicates	Suspected but unable to rate for narrative review	No downgrade	Funnel plot indicates	Suspected but unable to rate for narrative review	No downgrade
**Overall Judgement**			**Low Quality**			**Moderate Quality**			**Moderate Quality**
6. Dose-response	Significant trend	Yes	No upgrade	Significant trend	Limited	No upgrade	Significant trend	No	No upgrade
7. Magnitude of effect	RR > 2	Not assessed	No upgrade	RR > 2	Not assessed	No upgrade	RR > 2	Not assessed	No upgrade
8. Confounding adjusted	Effect in spite of confounding working towards the nil	Good control for confounding	No upgrade	Effect in spite of confounding working towards the nil	Good control for confounding	No upgrade	Effect in spite of confounding working towards the nil	Good control for confounding	No upgrade
**Overall Judgement**			**Low Quality**			**Moderate Quality**			**Moderate Quality**

**Table 9:** GRADE for the quality of evidence for wind turbine noise being associated with quality of life, wellbeing and mental health (5 systematic review studies).

Domains	Criterion	Assessment	Downgrading
Start Level	Longitudinal/Intervention	Cross-Sectional	Low
1. Study Limitations	Study quality & bias	Some studies low quality/high risk of bias	Downgrade
2. Inconsistency	Conflicting results; high I^2^	Inconsistent evidence; I^2^ not assessed. Small number of studies	Downgrade
3. Indirectness	Direct comparison; same PECCO	Indirect comparisons made.	Downgrade
4. Precision	Confidence interval contains 25% harm or benefit	Unable to rate for narrative review	No downgrade
5. Publication Bias	Funnel plot indicates	Suspected but unable to rate for narrative review	No downgrade
**Overall Judgement**			**Very Low Quality**
6. Dose-response	Significant trend	No	No upgrade
7. Magnitude of effect	RR > 2	Unable to assess	No upgrade
8. Confounding adjusted	Effect in spite of confounding working towards the nil	Some control for confounding but residual confounding likely to remain	No upgrade
**Overall Judgement**			**Very Low Quality**

## Supplementary Materials

The following are available online at http://www.mdpi.com/1660-4601/15/11/2400/s1. Tables S1–S7: data extraction tables for each of the outcome domains presented in this paper. Information on excluded papers are also provided.

## Appendix A. Search Terms

SEARCH TERMS: (note wind noise terms were included in initial search for systematic reviews but not in second search for individual papers) environmental noise; community noise; traffic noise; wind turbine noise; wind farm noise; wind turbine sound; wind farm sound; aircraft noise; airport noise; railway noise; road traffic noise; transportation noise; train noise; leisure noise; leisure-time noise; neighbourhood noise; neighborhood noise; household noise; low frequency noise; classroom noise; school noise; high-volume music; high-volume noise; noise from personal electronic devices; noise from mp3 players; noise from children’s toys; hospital noise; combined noise exposure; noise nuisance; noise exposure; truck noise; motor vehicle noise; noise load; entertainment noise; noise from mobile phones; noise from personal audio devices; noise from personal music players; combined exposure to noise and vibration; combined exposure to noise and air pollution; prospective and retrospective cohort studies, case-control studies and observational or experimental cross-sectional studies; self-reported quality of life (well-being, health status, vitality) using assessments such as the Short Form Health Survey (SF-36), General Health Questionnaire (GHQ), WHO Quality of Life assessment (WHOQOL and WHOQOL-BREF), Health-related Quality of Life (HRQOL); medication intake for treatment of anxiety and depression; self-reported depression, anxiety and psychological symptoms (scale); interview measures of depressive and anxiety disorders; hospital admission data for psychiatric disorders; emotional and conduct disorders in children (e.g., assessed by instruments such as strengths and difficulties questionnaire and KINDL); helplessness; behavioural/behavioral issues.

## Appendix B. Risk of Bias

**Reference**	**Bias due to Exposure Assessment**	**Bias due to Confounding**	**Bias due to Selection of Participants**	**Bias due to Health Outcome Assessment**	**Bias due to Not Blinded Outcome Assessment**	**Total Risk of Bias**
Barcelo Perez & Guzman Pineiro, Revista Cubana Hyg Epidemiol, 2008	High	High	High	Low	High	High
Belojevic, et al., J Environ Psychol, 2012	Low	Low	High	Low	Low	Unclear
Black et al., J Air Transp Manag, 2007	Low	High	Low	Low	Low	Low
Bocquier et al., Eur J Public Health, 2013	Low	Low	Low	High	Low	Low
Brink et al., Environ Int, 2011	Low	Low	Low	High	Low	Unclear
Clark et al., Am J Epidemol, 2012	Low	Low	Low	Low	Low	Low
Clark et al., J Enviro Psychol, 2013	Low	Low	Low	Low	Low	Low
Crombie et al., Enviro Health, 2011	Low	Low	Low	Low	Low	Low
Floud et al., Occup Environ Med, 2011	Low	Low	Low	Low	Low	Low
Fooladi, J Environ Public Health, 2012	High	High	High	High	High	High
Halonen et al., Scand J Work Environ Health, 2014	Low	Low	Low	Low	Low	Low
Hardoy et al., Soc Psychiatry Psychiatr Epidemiol, 2005	High	High	High	High	Low	High
Heritier et al., 2014	Low	Low	High	Low	Low	Unclear
Hjorteberg et al., Env Health Perspect, 2015	Low	Low	Low	Low	Low	Low
Honold et al., J Environ Psychol, 2012	Unclear	Unclear	High	Low	Low	High
Kishikawa, et al., Noise Health, 2009	Low	High	Unclear	Low	Low	High
La Torre et al., J Public Health, 2007	High	High	High	Low	Unclear	High
Roswall et al., PLOS One, 2015	Low	Low	Low	Low	Low	Low
Schreckenberg et al., Int J Environ Res Public Health, 2010	Low	Low	Unclear	Low	Low	Unclear
Schreckenberg et al., Noise & Health, 2010	Low	Low	Low	Low	Low	Low
Schreckenberg et al., NORAH study, 2015	Low	Low	High	Low	Low	Unclear
Stansfeld et al., JEP, 2009 RANCH	Low	Low	Low	Low	Low	Low
Stansfeld et al., Lancet, 2005	Low	Low	Low	Low	Low	Low
Stansfeld et al., Noise Health, 2009	Low	Low	Low	Low	Low	Low
Sygna et al., Environ Res, 2014	Low	Low	Low	Low	Low	Low
Tiesler et al., Enviro Res, 2013	High	High	High	High	High	High
Urban & Maca, Int J Environ Res Public Health, 2013	Low	High	Low	High	Low	High
Van Kempen et al., J Acoust Soc Am, 2010	Low	Low	Low	Low	Low	Low
Welch et al., Noise and Health 2013	High	High	High	Low	Low	High

In order to score ‘low’ for ‘bias due to confounding’ the study should at least adjust the analyses for age and sex. In order to score ‘low’ for bias due to selection of participants the participants had to be randomly sampled from a known population and the response rate of the study had to be >=60%.

## Appendix C

Supplemental material: list of excluded papers during the eligibility stage of data extraction.

The 21 papers excluded from the review after data extraction are listed below, along with the primary reason for the exclusion which included studies of an experimental design; studies with no mental health outcome or noise measurement/assessment; review articles; and primary research studies of wind-turbine and mental health, which were already covered by our evaluation of existing systematic reviews for this exposure and outcome:

1. Pawlaczyk-Luszczyniska, M.; Dudarewicz, A.; Waszkowska, M.; Szymczak, W.; Sliwińska-Kowalska, M. The impact of low-frequency noise on human mental performance. *Int. J. Occup. Med. Environ. Health* **2005**, *18*, 185–198. (Experimental study)
2. Bowling, A.; Barber, J.; Morris, R.; Ebrahim, S. Do perceptions of neighbourhood environment influence health? Baseline findings from a British survey of aging. *J. Epidemiol. Community Health* **2006**, *60*, 476–483. (No noise data/measurement)
3. Guite, H.F.; Clark, C.; Ackrill, G. The impact of the physical and urban environment on mental well-being. *Public Health* **2006**, *120*, 1117–1126. (No noise data/measurement)
4. Tomei, G.; Tecchio, F.; Zappasodi, F.; Ercolani, M.; Moffa, F.; Chiovenda, P.; Ciarrocca, M. Exposure to traffic noise and effects on attention. *Annali di Igiene Medicina Preventiva e di Comunita* **2006**, *18*, 507–519. (Italian—unable to translate)
5. Chiovenda, P. Pasqualetti, P.; Zappasodi, F.; Ercolani, M.; Milazzo, D.; Tomei, G.; Capozzella, A.; Tomei, F.; Rossini, P.M.; Tecchio, F. Environmental noise-exposed workers: event-related potentials, neuropsychological and mood assessment. *Int. J. Psychophysiol.* **2007**, *65*, 228–237. (Experimental study)
6. Pedersen, E.; Waye, K.P. Wind turbine noise, annoyance and self-reported health and well-being in different living environments. *Occup. Environ. Med.* **2007**, *64*, 480–486. (Wind-turbine study—systematic reviews available)
7. Persson, R.; Björk, J.; Ardö, J.; Albin, M.; Jakobsson, K. Trait anxiety and modeled exposure as determinants of self-reported annoyance to sound, air pollution and other environmental factors in the home. *Int. Arch. Occup. Environ. Health* **2007**, *81*, 179–191. (Wind-turbine study—systematic reviews available)
8. Mahendra Prashanth, K.V.; Sridhar, V. The relationship between noise frequency components and physical, physiological and psychological effects of industrial workers. *Noise Health* **2008**, *10*, 90–98. (Review)
9. Shepherd, D. McBride, D.; Welch, D.; Dirks, K.N.; Hill, E.M. Evaluating the impact of wind turbine noise on health-related quality of life. *Noise Health* **2011**, *13*, 333–339. (Wind-turbine study—Systematic reviews available)
10. Stansfeld, S.; Clark, C. *Mental Health Effects of Noise*, in *Encyclopedia of Environmental Health*; Nriagu, J.O., Ed.; Elsevier: Burlington, MA, USA, 2011; pp. 683–689. (Review)
11. Clark, C.; Sorqvist, P. A 3 year update on the influence of noise on performance and behavior. *Noise Health* **2012**, *14*, 292–296. (Review)
12. Nissenbaum, M.A.; Aramini, J.J.; Hanning, C.D. Effects of industrial wind turbine noise on sleep and health. *Noise Health* **2012**, *14*, 237–243. (Wind-turbine study—Systematic reviews available)
13. Stansfeld, S.A.; Clark, C.; Crombie, R. *Noise*; Oxford Library of Psychology, Oxford University Press: New York, NY, USA, 2012; pp. 700–390. (Review)
14. Evrard, A.S.; Khati, I.; Champelovier, P.; Lambert, J. Laumon, B. *Cardiovascular Effects of Aircraft Noise Near Paris-Charles de Gaulle Airport: Results from the Pilot Study of the DEBATS Research Program*; INTER-NOISE 2013, the 42st International Congress and Exposition on Noise Control Engineering, Innsbruk, Austria, 2013. (No mental health outcome)
15. Greiser, E.; Glaeske, G. Social and economic consequences of night-time aircraft noise in the vicinity of Frankfurt/Main airport. *Gesundheitswesen* **2013**, *75*, 127–133. (Burden of Disease/Economic study)
16. Hua, H.; Karlsson, J.; Widén, S.; Möller, C.; Lyxell, B. Quality of life, effort and disturbance perceived in noise: A comparison between employees with aided hearing impairment and normal hearing. *Int. J. Audiol.* **2013**, *52*, 642–649. (Experimental study)
17. Roosli, M. Health effects of environmental noise exposure. *Ther. Umschau Revue Ther.* **2013**, *70*, 720–724. (Review)
18. Tzivian, L.; Winkler, A.; Dlugaj, M.; Schikowski, T.; Vossoughi, M.; Fuks, K.; Weinmayr, G.; Hoffmann, B.Effect of long-term outdoor air pollution and noise on cognitive and psychological functions in adults. *Int. J. Hyg. Environ. Health* **2015**, *218*, 1–11. (Review)
19. Ristovska, G.; Gjorgjev, D. Assessment of Health Effects Related to Noise Exposure in Adult Population in Urban Center Skopje; In *39^th^ International Congress on Noise Control Engineering 2010, INTER-NOISE 2010;* Curran Associates, Inc.: Lisbon, Porgugal, 2011; pp. 6656–6662. (No noise measurement)
20. Halonen, J.I.; Vahtera, J.; Stansfeld, S.; Yli-Tuomi, T.; Salo, P.; Pentti, J.; Kivimäki, M.; Lanki, T. Associations between Nighttime Traffic Noise and Sleep: The Finnish Public Sector Study. *Environ. Health Perspect.* **2012**, *120*, 1391–1396. (No mental health outcome in relation to noise exposure. Focus is on insomnia and anxiety is only reported as a potential moderator of the association between noise and insomnia)

After peer-review for publication it was requested that the authors consider a number of papers for further inclusion in the review. Many of these papers had already been excluded at the screening stage. These are listed below along with reasons for their exclusion.

1. Fyhri, A.; Aasvang, G.M. Noise, sleep and poor health: modeling the relationship between road traffic noise and cardiovascular problems. *Sci. Total Environ.* **2010**, *408*, 4935–4942. (examines pseudoneurological complaints but only as a moderator of the association of noise on sleep disturbance)
2. Walinder, R.; Gunnarsson, K.; Runeson, R.; Smedje, G. Physiological and psychological stress reactions in relation to classroom noise. *Scand. J. Work. Environ. Health* **2007**, *33*, 260–266. (assesses internal noise not external environmental noise)
3. Riedel, N.; Kockler, H.; Scheiner, J.; Berger, K. Objective exposure to road traffic noise, noise annoyance and self-rated poor health—Framing the relationship between noise and health as a matter of multiple stressors and resources in urban neighbourhoods. *J. Environ. Plan. Manag.* **2015**, *582*, 336–356. (not identified in the searches undertaken)
4. De Kluizenaar, Y.; Janssenn, S.A.; van Lenthe, F.J.; Miedema, H.M.E.; Mackenbach, J.P. Long-term road traffic noise exposure is associated with an increase in morning tiredness. *J. Acoust. Soc. Am.* **2009**, *126*, 626. (examines tiredness which was scoped out of the self-rated health outcomes under-consideration in this review. Relevant for sleep disturbance review)
5. Yoon, J.H.; Won, J.U.; Lee, W.; Jung, P.K.; Roh, J. Occupational noise annoyance linked to depressive symptoms and suicidal ideation: a result from nationwide survey of Korea. *PLoS ONE* **2014**, *21*, 8. (occupational exposure—out of scope)
6. Wright, B.; Peters, E.; Ettinger, U.; Kuipers, E.; Kumari, V. Understanding noise stress-induced cognitive impairment in healthy adults and its implications for schizophrenia. *Noise Health* **2014**, *16*, 166–176. (review paper)
7. Oiamo, T.H.; Luginaah, N.; Baxter, J. Cumulative efects of noise and odour annoyances on environmental and health related quality of life. *Soc. Sci. Med.* **2015**, *146*, 191–203. (published after the search cut-off date of early October 2015)
8. Tabraiz, S.; Ahmad, S.; Shehzadi, I.; Asif, M.B. Study of physio-psychological effects on traffic wardens due to traffic noise pollution: exposure-effect relation. *J. Environ. Health Sci. Eng.* **2015**, *13*, 30. (occupational exposure—out of scope)
9. Greiser, E.; Greiser, C.; Jahnsen, K. Night-time aircraft noise increases prevalence of prescriptions of antihypertensive and cardivascular drugs irrespective of social class—The Cologne-Bonn Airport study. *J. Public Health* **2007**, *155*, 327–337. (examines anxiolytic medication in relation to medication for cardiac and antihypertensive medication: does not report the direct assocation between noise and anxiolytic medication)
10. Rudisser, J.; Lercher, P.; Heller, A. Traffic exposure and medication—A GIS based study on prescription of medicines in teh Tyrolean Wipptal. *Ital. J. Public Health* **2008**, 5, 261–267. (No noise assessment: measures distance to noise source)
