# Supplementary material for: WHO Environmental Noise Guidelines for the European Region: A Systematic Review on Environmental Noise and Quality of Life, Wellbeing and Mental Health
Source: Int J Environ Res Public Health. 2018 Oct 29;15(11):2400. doi: 10.3390/ijerph15112400 (PMC6266190; doi:10.3390/ijerph15112400)
Supplement: Supplementary file 1 [file ijerph-15-02400-s001.pdf]

Supplementary Table 1: Data extraction for environmental noise effects on self-reported quality of life or health

	Reference	**Population**: general population in settings (hospitals, residences, public venues, educational facilities) + **response rate** and other **selection** /bias factors **Cross-sectional or longitudinal**	**Exposure**: exposure to high levels of environmental noise from various noise sources + noise metric involved + modelled or measured noise	**Comparator**: no noise exposure or lower levels of noise exposure	**Confounding**: adjusted for confounding	**Outcome**: assessment of outcome	**Findings:** expressed as effect per dB if possible.<br><br>Type of analyses<br><br>Sample size relating to the effect size	**Comments:** Anything else to note
**AIRCRAFT NOISE EXPOSURE**								
**CHILD POPULATIONS**								
**CROSS-SECTIONAL EVIDENCE**								
1.	Clark et al, Am J Epidemol, 2012	**Population**: school children, total n=960, aged 9-10yrs. **Cross sectional** study	**Noise exposure**: aircraft noise levels at home and at school **measured** in an area from 7 am to 11 pm; road traffic noise at school **combined** from measurements and models **Noise source**: aircraft and road traffic **Noise metrics**: LAeq, 16h (dB)	**Comparator**: lower levels of noise exposure	**Confounding**: Modelled concentrations of **NO2** (µg/m3); age, gender, socio-economic status (employment, home ownership, home crowding), ethnicity, maternal education, main language spoken at home, long-standing illness, parental support for schoolwork, classroom glazing	**Outcome**: **Perceived child's health** – very good/good vs. fair/poor/very poor, perceived by parents; **Psychological distress** measured using Strengths and Difficulties Questionnaire (Goodman, 1997), fulfilled by parents	**Findings:** No association between aircraft and road traffic noise and self-rated health or psychological distress **Type of analyses:** Multilevel linear and logistic regression models **Sample size relating to the effect size:** n=719 with air pollution data and n=241 without air pollution data	**Comments:** **Findings:** No association between aircraft and road traffic noise and systolic and diastolic blood pressure
2.	Van Kempen et al, J Acoust Soc	**Population**: school children, 89 schools	**Noise exposure**: aircraft noise	**Comparator**: lower levels	**Confounding**: age, gender,	**Outcomes:** **Perceived health**	**Findings:** Children **annoyed**	**Comments:** **Findings:**

	Am, 2010	around three airports, total n=2844, aged 9-11yrs.<br>**Cross sectional** study	levels at home and at school **measured** in an area from 7 am to 11 pm; road traffic noise at school **modelled** or **combination** of measurements with models<br>**Noise source**: aircraft and road traffic<br>**Noise metrics**: LAeq, 16h (dB)	of noise exposure	country, socio-economic status (home crowding, home ownership, employment, mother's education), ethnicity, long-standing illness, main language spoken at home, parental support for school work, other noise exposure, annoyance	– self-reported health symptoms (headache, vomiting, stomachache, difficulty falling asleep, being woken at night or feeling sleepy during a day);<br>**Neurobehavioral functioning –** Neurobehavioral Evaluation System (NES) (Letz, 1991), including the following: Simple Reaction Time Test (SRTT), Switching Attention Test (SAT), Hand-Eye Coordination Test (HECT), Symbol Digit Substitution Test (SDST), Digit Memory Spin Test (DMST);<br>**Aircraft and road traffic noise annoyance** measured on 5-point scale (ISO 2003) fulfilled by children	**by aircraft or road traffic** noise at school reported significantly more symptoms compared to children who were not annoyed. After pooling the data, the association was no longer evident.<br>Children **annoyed by aircraft** noise at school made significantly more faults at the switch condition of SAT test and had shorter span length on DMST test, compared to not-annoyed children.<br>**Type of analyses:** Multilevel modelling analyses<br>**Sample size relating to the effect size:** n=553 with complete NES data, n=2844 with complete health symptoms data	No association between aircraft and road traffic noise and number of self-reported health symptoms.<br>No differences between children annoyed by road traffic noise and not-annoyed children in any NES tests.<br>**Conclusion:** The association between noise and health and neurobehavioral functioning is not confounded by annoyance.
3.	Stansfeld et al, Lancet, 2005	**Population**: school children, 89 schools	**Noise exposure**: aircraft noise	**Comparator**: lower levels	**Confounding**: age, gender,	**Outcomes: Self-reported**	**Findings:** Increase of **aircraft**	**Comments: Findings:**

		around three airports, total n=2844, aged 9-13yrs.<br>**Cross sectional** study	levels at home and at school **measured** in an area from 7 am to 11 pm; road traffic noise at school **modelled** or **combination** of measurements with models<br>**Noise source**: aircraft and road traffic<br>**Noise metrics**: LAeq, 16h (dB)	of noise exposure	parental employment, home ownership, home crowding, mother's educational attainment, long-standing illness, main language spoken at home, parental support for school work, classroom glazing	**health** – perceived by children;<br>**Psychological distress** – perceived children's mental health, measured using Strengths and Difficulties Questionnaire (Goodman, 1997), fulfilled by parents<br>**Aircraft noise annoyance** measured by 5-point scale (ISO 2003) fulfilled by children	noise at school by 1 dB was significantly associated with an increase of noise annoyance by 0.037 marks.<br>Increase of **road traffic** noise at school by 1 dB was significantly associated with an increase of noise annoyance by 0.016 marks.<br>**Type of analyses:** Multilevel model analyses (for data clustering)<br>**Sample size relating to the effect size:** range from 1939 to 2014 with complete data	No association between aircraft and road traffic noise and self-reported health, and perceived children's mental health.
**ADULT POPULATIONS**								
**LONGITUDINAL EVIDENCE**								
4.	Schreckenberg et al., NORAH study, 2015	**Population**: Four airports in Germany Frankfurt/Main, Koln/Bonn, Stuttgart, Berlin/Brandenburg; airport; adults: total n=14959 eligible persons, response rate 7-31% (by area), age 18 to above 80 years<br>**Cross-sectional**	**Noise exposure:** Noise contours around airports; alone or combined with road-traffic, rail-traffic<br>**Noise source:** aircraft noise<br>**Noise metrics:** LAeq_day,	**Comparison:** Change estimate per 5 dBA increase of Leq_24h	**Confounding:** Age, gender, migrant background. Social factors: adjustment to traffic, expectations for traffic development, expectations for airport use, trust in the efforts of	**Outcomes: Health-related quality of life** – measured with SF-8 scale (short version of SF-36, Ellert et al, 2005); two sub-components defined: physical and mental quality of life (Quality Metrics,	**Findings: Cross-sectional study:** significant negative correlation between mental and physical quality of life scores and Leq_24h at all four airports.<br>**Follow-up study:** following the	**Comments: Findings:** Significant interaction of age, female gender, BMI, socio-economic status, physical activity, noise sensitivity and noise annoyance on the association

		study (in 2011)<br>**Follow-up study**<br>2012-2013 after the opening of a new runway in Frankfurt/Main airport	LAeq_night, Leq_24h, Lden<br>**Noise groups:**<br>10 classes of noise levels in range 40-65 dB (by 2.5 dB) for cross-sectional study; noise levels changes: increase > + 2 dB, decrease > - 2 dB or stable levels for the follow-up study		those reponsible for noise regulation, fairness.<br>Personal factors: noise coping strategies, noise sensitivity (short form 12-item NoiSeQ-Reduced), physcial activity, smoking, alcohol intake, BMI.<br>Dwelling characteristics, exposure to other noise sources.	2011)<br>**Other outcomes**:<br>Noise annoyance, sleep disturbances, disturbance of everyday activities, psycho-vegetative disturbances, residential satisfaction	reduction of noise levels, mental quality of life increased in groups exposed to noise levels ≥50 dBA; physical quality of life decreased in almost all noise exposure groups.<br>Following the increase of noise levels, mental quality of life decreased only in group exposed to 40-45 dBA; following the reduction of noise levels, physical quality of life was not significantly changed.<br>A significant correlation between mental and physical quality of life with noise annoyance.<br>**Type of analyses:**<br>Correlation, inferential analysis, multilevel regression<br>**Sample size relating to the effect size:**	between aircraft noise levels and mental and physical quality of life in all airports.

							n=9244 cross-sectional; n=4867 (1-year follow-up), n=3508 (2-year follow-up) in Frankfurt/Main study	
**CROSS-SECTIONAL EVIDENCE**								
5.	Black et al, J Air Transp Manag, 2007	**Population**: adult residents from a suburb area near the airport and control area matched for socio-economic status, total n=704, aged 15-87yrs. **Selection**: n=1500 estimated sample size, n=796 returned questionnaire, n=704 filled the data, response rate 50% **Cross sectional** study	**Noise exposure**: aircraft noise levels **measured** in the area from 7 a.m. to 6 p.m. **Noise source**: aircraft noise **Noise metrics**: LAeq 7am-6pm (dB) **Noise groups**: aircraft noise and control group	**Comparator**: Aircraft noise exposed area compared to control area	**Confounding**: Noise sensitivity, traffic noise annoyance, aircraft noise annoyance	**Outcomes: Health-related quality of life** – assessed with SF-36 (Ware & Sherbourne, 1992), including physical functioning, general health, vitality and mental health; **Noise annoyance** from road traffic and aircraft noise – not explained; **Noise stress**– not explained; **Noise sensitivity** – assessed by modified Weinstein scale (Weinstein, 1978)	**Findings:** Persons exposed to **aircraft noise** had significantly lower scores on mental health subscale in comparison to persons from the control area. Persons exposed to aircraft noise had higher levels of noise annoyance and noise stress, but similar noise sensitivity. **Type of analyses:** Logistic regression model **Sample size relating to the effect size:** n=704	**Comments: Findings:** Chronic noise stress was significantly predicted by noise exposure group, noise sensitivity, traffic annoyance and aircraft annoyance.
6.	Schreckenberg et al, Int J Environ Res Public Health, 2010	**Population**: adult residents living near an airport, total n=2312, aged 17 to >80yrs. **Selection**: n=3795 eligible, n=2312	**Noise exposure**: aircraft noise exposure, road traffic and railway noise were **modelled** at daytime and	**Comparator**: lower levels of noise exposure	**Confounding**: Age, gender, house ownership, socioeconomic status (income, education, occupational	**Outcomes: Health-related quality of life** – measured with SF-36 and SF-12 scales (Bullinger & Kirchberger	**Findings: Aircraft noise** annoyance and noise sensitivity (but not aircraft noise levels) were significant predictors of poor	**Comments: Findings:** Aircraft Lden correlated significantly and positively with noise annoyance,

		returned the questionnaire, response rate 61% **Cross sectional** study	nighttime **Noise source**: aircraft **Noise metrics**: LAeq 16h daytime (6 a.m. and 10 p.m.), Leq 8h night (10 p.m. to 6 a.m.), Ldn, Lden (dB)		status), noise sensitivity, residential satisfaction, usual window position in the sleeping room at night, number of hours away from home, railway and road traffic sound level, morbidity	1998); **Health complaints** – self-reported scale (GSCL-24) (Braehler et al, 2008); **Residential satisfaction** – assessed with 5-point scale, including dwelling, residential area, infrastructure (Wirth, 2000); **Attitudes related to air traffic –** a self-reported scale; **Noise annoyance** – assessed on a 5-point verbal and a 11-point numerical scale; **Coping with aircraft noise** – measured on a 5-point frequency scale; **Noise sensitivity** – assessed from one question; **Sleep quality** – assessed by the Pittsburgh Sleep Quality Index (PSQI) (Buysse et al, 1989)	health-related quality of life, health complaints and poor sleep quality. **Type of analyses:** Linear correlation, logistic regression models, multi-factorial general linear model **Sample size relating to the effect size:** n=2311 with complete data	disturbance of activities, coping strategies, negative expectations, fears related to aircraft, and negatively with residential satisfaction parameters. Pre-existing health problems (multi-morbidity) may moderate the impact of aircraft noise exposure on health-related quality of life.

7.	Schreckenberg et al, Noise & Health, 2010	**Population**: adult residents living near an airport, total n=190, aged 17-80yrs. **Selection**: n=2310 eligible, n=190 with noise sensitivity data **Cross sectional** study	**Noise exposure**: aircraft noise and road traffic noise were **modelled** at daytime and nighttime **Noise source**: aircraft and road traffic **Noise metrics**: LAeq 16h daytime (6 a.m. and 10 p.m.) (dB)	**Comparator**: lower levels of noise exposure	**Confounding**: Age, gender, social status (income, education, and occupational status)	**Outcomes:** **Noise sensitivity** – assessed from 35-item questionnaire (NoiSeQ) (Schütte et al, 2007); **Noise annoyance** - due to aircraft and road traffic noise, assessed on a 5-point verbal scale (Fields, 2001); **Environmental and social problems –** using a 23-item scale; **Residential satisfaction** – assessed with 16-item scale (Wirth, 2000); **Health-related quality of life** – measured with SF-12 scale (Bullinger & Kirchberger 1998); **Diagnosed diseases** – self-reported scale (Bellach et al, 1998); **Life satisfaction** – measured with FLZ-A scale (Herschbach &	**Findings:** **Aircraft and road traffic noise** annoyance and aircraft noise level at daytime were negatively correlated with residential satisfaction, but not with health-related quality of life. Noise annoyance (aircraft and road traffic) was significantly predicted by daytime Leq 16h (respectively). **Aircraft noise levels** were associated with higher perception of global environmental problems, and with lower global residential satisfaction. **Type of analyses:** Linear correlation, linear multiple regression analysis **Sample size relating to the effect size:** n=163-190 with complete data	**Comments:** **Findings:** Noise sensitivity was predicted by age and reported physical health, but not with reported mental health. Noise sensitivity was direct predictor of total and aircraft noise annoyance, higher perception of environmental and social problems, and predictor of residential satisfaction (lower quietness). **Conclusion:** Noise sensitivity is not part of a general tendency to be more aware of the negative aspects of one's environment.

						Henrich 1991); **Sleep quality** – assessed by the Pittsburgh Sleep Quality Index (PSQI) (Backhaus et al, 2001)		
**ROAD TRAFFIC NOISE**								
**CHILD POPULATIONS**								
**CROSS-SECTIONAL EVIDENCE**								
	See Clark et al, Am J Epidemol, 2012							
	See Van Kempen et al, J Acoust Soc Am, 2010							
	See Stansfeld et al, Lancet, 2005							
**ADULT POPULATIONS**								
**LONGITUDINAL EVIDENCE**								
8.	Heritier et al, 2014	**Population:** adults from Basel Switzerland. Response rate cross-sectional 37% (n=1375); one-year follow up 82% (n=1172)	**Noise Exposure:** Modelled noise exposure geocoded. Road traffic for 2007. Time-weighted daily average levels. Ldn calculated for road traffic noise including the 10dBA penalty for the night-time.	Comparator: Estimates for 10dB increase in Ldn.	**Confounding:** age, gender, physical activity, smoking, educational level, marital status, urban/suburban region.	**Outcomes:** SF-36 and von Zerssen symptom score. Self-reported health status.	**Findings:** a 10 dB(A) increase of the road traffic noise Ldn was associated with a 0.47 (95% CI: −0.01, 0.95, p=0.05) point increase of the von Zerssen symptom score. Borderline significant.<br><br>No association between a 10dB(A) increase in road traffic noise Lden and SF36 (0.09	**Comments:** Low response rate for the original survey. Self-reported health outcomes. No adjustment for noise sensitivity.<br><br>It isn't clear if some of the findings are cross-sectional, longitudinal or combine both time-points for analysis.

							(95%CI -0.43, 0.61), p=0.73).<br><br>A 10dB(A) incrase in road traffic noise Ldn was associated with a decrease in self-reported health status OR=1.28, 95%CI 1.12-1.48), p=0.001.<br><br>Confirm association of noise with self-reported health and no associations between noise and von Zerssen symptom score and SF36.	
**INTERVENTION EVIDENCE**								
9.	Stansfeld et al, Noise Health, 2009	**Population**: adult residents living in noisy and quiet areas, total n=387, aged 16-90yrs.<br>Response rate at baseline 70%, at follow-up 74%<br>**Longitudinal /Intervention** study, baseline in 1997, n=337, follow-up in 1998, n=228 after the opening of a bypass road (change of noise level by 2-4 dBA)	**Noise exposure**: road traffic noise levels **measured** between 10 am and 5 pm on weekdays;<br>**Noise source**: road traffic<br>**Noise metrics**: Leq, L10 (dBA)<br>**Noise groups**: noise exposed group (facing the main street, L_{10}=75-78 dBA), control group (on	**Comparator**: Noise exposed group compared to control group; within-subjects comparison	**Confounding**: Deprivation index (Townsend's scale including: car ownership, home ownership, crowding, unemployment), baseline health, age, sex	**Outcomes:**<br>**Health status** – SF-36 General Health Survey (Ware & Sherbourne 1992);<br>**Psychological distress** – General Health Questionnaire (GHQ), 28 items (Goldberg & Hillier, 1979);<br>**Prevalence of mental disorders** – assessed by the Revised Clinical Interview	**Findings:**<br>At baseline, people from **high and low noise groups** had similar health status. At follow-up no differences in health status between the groups.<br>At follow-up, after the bypass opened, there was a decrease in prevalence rates of mental disorder in both groups.<br>**Type of analyses:** Univariate analyses of covariance	**Comments:**<br>**Findings:**<br>At follow-up, there was a decrease in noise annoyance in the high noise group.

			uncongested side streets, L_{10}=55-58 dBA)			Schedule (Lewis et al, 1992); **Noise annoyance** – from neighbors, road traffic and train noise (Fields, 1992)	(ANCOVA), Univariate analyses of covariance adjusted for baseline health status **Sample size relating to the effect size:** n=387 with complete data; n=71 interviewed for prevalence of mental disorder	
**LONGITUDINAL EVIDENCE**								
10	Roswall et al, PLOS One, 2015	**Population**: adult residents in greater urban area, n=38,964, aged 50-64 yrs. **Selection**: n=45,271 eligible of the original cohort (79%), n=5662 excluded due to missing data **Nationwide cohort** study	**Noise exposure**: road traffic and railway noise were **modeled** at daytime and nighttime **Noise source**: road traffic and railway **Noise metrics**: road traffic: Lden (dBA); Railway noise: categories 0 dB, <55 dB, ≥55 dB	**Comparator**: lower levels of noise exposure (per 10 dBA)	**Confounding**: Age, sex, education, cohabiting status, income, Charlson Comorbidity Index, railway noise exposure, smoking status, waist circumference, alcohol intake	**Outcomes: Health-related quality of life** – measured with SF-36 scale-Danish (Maruish, 2011); two summary scales: physical component summary (PCS) and mental component summary (MCS)	**Findings: Road traffic noise** exposure 1 year preceding the testing was associated with a decrease in both PCS (by 0.32 points) and MCS (by 0.42 points) – crude models. Similar results for 10 year noise exposure. After adjustment for socioeconomic factors, a 10 dBA increase in road traffic noise was associated with a decrease of MCS (by 0.14 points for 1 year exposure; by 0.15 points for 10 yrs noise exposure.	**Comments: Findings:** No association of road traffic noise exposure with physical or mental component summaries when individual lifestyle covariates are taken into account (smoking, alcohol intake, waist circumference).

							**Type of analyses:** Linear regression models<br>**Sample size relating to the effect size:** n=38,964 with complete data	
**CROSS-SECTIONAL EVIDENCE**								
11	Barcelo Perez & Guzman Pineiro, Revista Cubana Hyg Epidemiol, 2008	**Population**: housewives living in an urban area in one city, total n=133, mean age 31yrs.<br>**Inclusion criteria**: age range 20-40yrs, unemployed, literacy<br>**Cross sectional** study	**Noise exposure**: road traffic noise levels **measured** for 1 hour at daytime in a typical urban area<br>**Noise source**: road traffic<br>**Noise metrics**: Leq, 1 hour (dB A, F)	**Comparator**: None	**Confounding**: Age, length of residence, education, level of profession, urban characteristics	**Outcomes:**<br>**Noise annoyance** –self-reported 5-point scale (no reference);<br>**Perceived stress test** – 10-item scale (Cohen, 2008);<br>**Health status** – abbreviated form (11-items) of SF-36 test (Ware, 2005);<br>**Perception of noise** – self-reported	**Findings:**<br>Noise annoyance was associated with medical history, urban characteristics, education level and occupation. It was not related with noise levels.<br>Health status was associated with age, length of residence.<br>Noise perception was associated with education, length of residence, not with noise levels.<br>**Type of analyses:** Linear regression analysis<br>**Sample size relating to the effect size:** n=133	**Comments:**<br>The method is observational, no comparisons, no correlation with noise exposure.
12	Halonen et al, Scand J Work Environ Health, 2014	**Population**: adult residents from a cohort study in three towns, total n=15 611, aged 21-76yrs.<br>**Cross sectional** study	**Noise exposure**: road traffic noise levels **modeled** for the streets<br>**Noise source**:	**Comparator:** Five noise level groups	**Confounding:** Trait anxiety as an indicator of noise sensitivity; age, sex, occupational status, education	**Outcomes:**<br>**Self-rated health** – using a 5-point scale;<br>**Psychotropic medication use** –	**Findings:**<br>Men exposed to **road traffic noise** >60 dBA had poorer self-rated health compared to men	**Comments:**<br>**Findings:**<br>No association between noise exposure and use of psychotropic

			road traffic<br>**Noise metrics**: Lden (dBA)<br>**Noise groups**: ≤45 dB (n=2821), 45.1–50 dB (n=4110), 50.1–55 dB (n=3597), 55.1–60 dB (n=2445), >60 dB (n=2638)		level, size of apartment, job strain, smoking, leisure-time physical activity, obesity, heavy alcohol drinking, chronic illness, area-level socio-economic status, population density	from National Prescriptions Register, including anxiolytics, antidepressants, and hypnotics; **Trait anxiety score** – based on a 6-item inventory	exposed to ≤45 dBA. The association was significant even after adjustment for trait anxiety, smoking, alcohol, obesity, physical inactivity.<br>**Type of analyses:** Logistic regression models<br>**Sample size relating to the effect size:** n=15611 with complete data	medication in men and women.
13	Honold et al, J Environ Psychol, 2012	**Population**: adult residents living in high noise, high air pollution area, total n=428, aged 16-91yrs.<br>**Selection**: n=2000 approached, n=428 returned, response rate 21.4%<br>**Cross sectional** study	**Noise exposure**: road traffic noise levels **modeled** in the area;<br>**Noise source**: air traffic<br>**Noise metrics**: not specified, probably Leq,24h (dBA)<br>**Noise groups**: High-burden blocks (high noise >65 dBA, high air pollution), low-burden blocks (noise ≤50 dBA)	**Comparator:** High-burden group compared to low-burden group	**Confounding**: Gender, age, educational attainment, nationality, marital status, employment, occupational position, size of community, use of medications, housing conditions	**Outcomes:**<br>**Neighborhood satisfaction** – using a 4-item scale (Feuersenger, 2004); **Life satisfaction** – assessed using an 5-item scale (Diener et al, 1985);<br>**Health behavior** – smoking, alcohol intake, physical activity (Feldman & Steptoe, 2004);<br>**General physical health** – one item from the SF-36 health survey (Bullinger &	**Findings:**<br>People from **high burden blocks** had significantly lower neighborhood satisfaction, and poorer health behavior.<br>Poor health behavior was related to employment, perceived traffic noise and perceived air quality.<br>**Type of analyses:** Analysis of variance (ANOVA), logistic regression, analysis of covariance (ANCOVA), hierarchical regression analysis<br>**Sample size**	**Comments:**<br>**Findings:**<br>No difference in life satisfaction, general physical health, anxiety and depression between high-burden and low-burden groups.<br>**Perceived air quality** was related to life satisfaction, general health, anxiety and depression.

						Kirchberger, 1998); **Depression and anxiety** – items on the Brief Symptom Inventory (Franke, 2000); **Environmental perception** – six factors, 5-point scale; **Environmental stress appraisal /annoyance** – four factors, 5-point scale	**relating to the effect size:** n=215 from high-burden group, n=213 from low-burden group	
14	Kishikawa, et al, Noise Health, 2009	**Population**: adult residents living along truck roads, total n=323, aged 20-70yrs. **Selection**: n=486 approached, response rate 88.2%, exclusion: aged over 70yrs. **Cross sectional** study	**Noise exposure**: road traffic noise levels at home **measured** in the area for 24 hours; **Noise source**: traffic on the trunk roads **Noise metrics**: Ldn (dBA) **Noise groups**: <55 dBA, 55-65 dBA, >65 dBA	**Comparator**: lower levels of noise exposure	**Confounding**: Age, gender, socioeconomic status, noise sensitivity (sensitive vs. insensitive)	**Outcomes: Subjective health** – using General Health Questionnaire GHQ-28 (Goldberg, 1978) with four subscales (somatic symptoms, anxiety & insomnia, social dysfunction, severe depression); **Noise sensitivity** – using original WNS scale (Weinstein, 1978), and a modified scale (WNS-6B); **Disturbances of**	**Findings:** Among noise sensitive persons, the odds ratios for overall GHQ scale, somatic symptoms and anxiety and insomnia increased significantly with Ldn. Among noise-insensitive persons the odds ratio for social dysfunction decreased with Ldn. **Type of analyses:** Multiple logistic regression models **Sample size relating to the effect size:** n=323 with	**Comments: Findings:** Among noise-sensitive persons in the highest noise exposure group (>65 dBA), the odds ratio for psychiatric cases on GHQ scale increased with sleep disturbance – sleep disturbance a possible modifier between noise exposure, sensitivity and psychiatric cases.

						**daily life** – sleep disturbance, speech interference, disturbance of watching TV (all assessed using a 5-point scale)	complete data	
15	La Torre et al, J Public Health, 2007	**Population**: adult residents living in noisy area, total n=159, aged <18 to >50yrs. **Cross sectional** study	**Noise exposure**: road and railway traffic noise levels **measured** at each point of the selected area three times for a week; **Noise source**: road and railway traffic **Noise metrics**: Average L (dBA) **Noise groups**: ≤65 dBA, >65 dBA	**Comparator**: High acoustic exposure group (>65 dBA) compared to low acoustic exposure group (≤65 dBA)	**Confounding**: Age, gender, educational level, hours spent at home, acoustic exposure level	**Outcomes: Health status** – Short-Form 36 item questionnaire (Apolone & Mosconi 1997), with four subscales: general health, vitality, emotional role and status, and mental health	**Findings:** People with **high acoustic exposure** had a significantly lower (worse) score on the mental health scale in comparison to low exposure level. High exposure is inversely associated with mental health scores in women only (adjusted). **Type of analyses:** Multiple linear regression models **Sample size relating to the effect size:** n=159 with complete data	**Comments: Findings:** People with high education level had significantly higher (better) scores on the general health scale, mental health subscale and emotional role subscale. Age and hours spent at home were inversely associated with all subscales.
	See Schreckenberg et al, Noise & Health, 2010							
16	Welch et al, Noise Health, 2013	**Population**: adult residents living near motorways, control area matched for	**Noise exposure**: road traffic noise levels **modelled** on	**Comparator:** Motorway group compared to	**Confounding**: Area of dwelling, noise sensitivity	**Outcomes: Health-related quality of life** – short form of	**Findings:** People from **motorway area** had lower scores on total	**Comments: Findings:** Noise sensitivity significantly

		socio-demographics, total n=502, aged 18 to >70yrs.<br>**Selection**: n=1250 approached, n=502 returned, response rate 40.1%<br>**Cross sectional** study	motorways based on traffic count;<br>**Noise source**: road traffic<br>**Noise metrics**: Ldn (dBA)<br>**Noise groups**: Motorway group (Ldn 75-77 dBA), non-motorway group (Ldn 50-61 dBA)	non-motorway group		WHO scale (WHOQOL), including subscales: physical, psychological, social, environmental;<br>**Neighborhood satisfaction - Noise sensitivity** – single question, agreement;<br>**Noise sensitivity** – self-rated into three categories (none, moderate, severe);<br>**Noise annoyance** – from road traffic and railway traffic (Fields, 1993);<br>**Annoyance by traffic fumes** – self-rated	quality of life scale and all subscales.<br>**Noise annoyance** and annoyance from traffic fumes were significantly related to lower quality of life on all subscales in the whole sample and in the motorway group.<br>**Type of analyses:** Spearman's and Pearson's correlation, analysis of variance (ANOVA)<br>**Sample size relating to the effect size:** n=257 from high-noise area, n=245 from low-noise area	modified the association between quality of life and area of dwelling.
17	Brink 2011, Environment International	**Population**: adult residents from a household survey, n=8261, mean age 45 yrs.<br>**Nationwide cohort**: Swiss Household Panel survey on living conditions, cases collected from 2004 to 2007<br>**Cross-sectional**	**Noise exposure**: road traffic, railway, aircraft noise were **modeled** at daytime and nighttime<br>**Noise source**: road traffic and railway<br>**Noise metrics**: Lday, Lnight, Ldn (dBA)	**Comparator**: lower levels of noise exposure (per 5 dBA)	**Confounding**: Age, sex, body mass index, socioeconomic status, financial satisfaction, satisfaction with living situation, satisfaction with personal relationships, region of residence, Swiss	**Outcomes: Subjective health status** – self-rated 5-point Likert scale;<br>**Satisfaction with health status** – self-rated 5-point Likert scale	**Findings: Road traffic and railway noise** exposure are significantly related to subjective health only in unadjusted models.<br>**Road traffic noise** exposure is significantly related to	**Comments: Findings:** Age and sex are the strongest determinants of subjective health; the satisfaction with personal relationships and the financial satisfaction are

					nationality		satisfaction with health status only in unadjusted models.<br>**Type of analyses:** Hierarchical linear regression models<br>**Sample size relating to the effect size:** n=8261 with complete data; n=8247 exposed to road traffic noise, n=4685 exposed to railway noise, n=499 exposed to aircraft noise	related with subjective health.
**RAILWAY NOISE**								
**LONGITUDINAL EVIDENCE**								
	See Roswall et al, PLoS One 2015 above							
**CROSS-SECTIONAL EVIDENCE**								
	See La Torre et al, J Public Health, 2007 above							
	See Brink Environment International 2011 above							

Supplementary Table 2: Data extraction for environmental noise effects on medication intake for the treatment of anxiety and depression

	Reference	**Population**: general population in settings (hospitals, residences, public venues, educational facilities) + **response rate** and other **selection** /bias factors **Cross-sectional or longitudinal**	**Exposure**: exposure to high levels of environmental noise from various noise sources + noise metric involved + modelled or measured noise	**Comparator**: no noise exposure or lower levels of noise exposure	**Confounding**: adjusted for confounding	**Outcome**: assessment of outcome	**Findings:** expressed as effect per dB if possible.<br><br>Type of analyses<br><br>Sample size relating to the effect size	**Comments:** Anything else to note
**AIRCRAFT NOISE EXPOSURE**								
**ADULT POPULATIONS**								
**CROSS-SECTIONAL EVIDENCE**								
1.	Floud et al, Occup Environ Med, 2011	**Population**: adult residents living near 7 airports in six countries, total n=4861, aged 45-70yrs.<br>**Inclusion**: length of residence ≥5yrs in six samples (≥3yrs in one sample)	**Noise exposure**: aircraft and road traffic noise levels **modelled** in the area at daytime (6 am-10 pm or 7 am-11 pm) and at night (10 pm-6 am or 11 pm-7 am)<br>**Noise source**: aircraft and road traffic	**Comparator:** Leq,16h and Lnight 10 dBA intervals	**Confounding:** Age, gender, body mass index, alcohol intake, physical activity, education, smoking, aircraft noise annoyance	**Outcomes: Prescribed medication use** – self-reported use in the previous 2 weeks, including anxiolytics, hypnotics, anti-depressants, antacids, anti-hypertensives, anti-asthmatics; **Noise annoyance**	**Findings:** The use of anxiolytics was significantly related to **aircraft** noise, both with Ldaytime (1.28 times higher per 10 dBA) and with Lnight (1.27 times higher per 10 dBA increase).<br>People **annoyed by aircraft noise** in day/at night had higher	**Comments:**<br>**Findings:** Some differences in the association between aircraft noise and the use of hypertensive drugs reported between the countries (the highest in UK, the lowest in Italy).

		**Cross sectional** study	**Noise metrics**: aircraft noise: Leq,16h daytime, Lnight (dBA); road traffic noise: Leq,24h **Noise groups**: aircraft noise: cut-off for Ldaytime 35 dBA, for Lnight 30 dBA; road traffic noise: cut-off 45 dBA			– by aircraft and road traffic noise, using 11-point numerical scale (ISO/TS 15666: 2003)	odds of taking: anti-hypertensives, anxiolytics or hypnotics (group), anxiolytics only, anti-depressants. **Type of analyses:** Hierarchical multilevel logistic regression, Spearman's correlation **Sample size relating to the effect size:** n=4641-4646 with complete data	No association between road traffic noise and use of any medication. Positive (but non-significant) association between road traffic noise and the use of antihypertensives or antacids in men.
**ROAD TRAFFIC NOISE**								
**ADULT POPULATIONS**								
2.	Bocquier et al, Eur J Public Health, 2013	**Population**: adult residents from a cohort study in one city, total n=190 617, aged 18-64yrs. **Cross sectional** study	**Noise exposure**: road traffic noise levels **modeled** in the city **Noise source**: road traffic at nighttime **Noise metrics**: Lnight (dBA) **Noise groups**: <45 dBA (21.4% of population), 45–50 dBA (31.6%), 50–55 dBA (35.2%), >55 dBA (11.8%)	**Comparator**: Lnight 5 dBA intervals	**Confounding**: Deprivation level calculated from: individual characteristics (sociodemographic, consultations with GP, chronic psychiatric disorder), char. of prescribers (demographic, specialty, work load), and neighborhood char. (medical density, complaints filed for noise)	**Outcomes: Anxiolytic-hypnotic drug use** – from National Health Insurance Fund, classified as N05B, N05CD and N05CF	**Findings:** Among persons from the low deprivation stratum, the risk of purchasing higher numbers of anxiolytics–hypnotics was higher only for Lnight levels >55 dB(A) (adjusted model). **Type of analyses:** Kruskal-Wallis test, Zero-inflated negative binomial (ZINB) model **Sample size relating to the effect size:** n=190 617	**Comments: Findings:** The proportion of persons exposed to high night-time noise levels increased with deprivation level.
3.	Halonen et al, Scand J Work	**Population**: adult residents	**Noise exposure**: road traffic noise	**Comparator:** Five noise	**Confounding:** Trait anxiety as an	**Outcomes: Self-rated health**	**Findings:** Men exposed to **road**	**Comments: Findings:**

	Environ Health, 2014	from a cohort study in three towns, total n=15 611, aged 21-76yrs. **Cross sectional** study	levels **modeled** for the streets **Noise source**: road traffic **Noise metrics**: Lden (dBA) **Noise groups**: ≤45 dBA (n=2821), 45.1–50 dBA (n=4110), 50.1–55 dBA (n=3597), 55.1–60 dBA (n=2445), >60 dBA (n=2638)	level groups	indicator of noise sensitivity; age, sex, occupational status, education level, size of apartment, job strain, smoking, leisure-time physical activity, obesity, heavy alcohol drinking, chronic illness, area-level socio-economic status, population density	– using a 5-point scale; **Psychotropic medication use** – from National Prescriptions Register, including anxiolytics, antidepressants, and hypnotics; **Trait anxiety score** – based on a 6-item inventory	**traffic noise** >60 dBA had poorer self-rated health compared to men exposed to ≤45 dBA. The association was significant even after adjustment for trait anxiety, smoking, alcohol, obesity, physical inactivity. **Type of analyses:** Logistic regression models **Sample size relating to the effect size:** n=15611 with complete data	No association between noise exposure and use of psychotropic medication in men and women.

Supplementary Table 3: Data extraction for associations of environmental noise exposure and self-reported anxiety and depression

	Reference	**Population**: general population in settings (hospitals, residences, public venues, educational facilities) + **response rate** and other **selection** /bias factors **Cross-sectional or longitudinal**	**Exposure**: exposure to high levels of environmental noise from various noise sources + noise metric involved + modelled or measured noise	**Comparator**: no noise exposure or lower levels of noise exposure	**Confounding**: adjusted for confounding	**Outcome**: assessment of outcome	**Findings:** expressed as effect per dB if possible.<br><br>Type of analyses<br><br>Sample size relating to the effect size	**Comments:** Anything else to note
**ROAD NOISE EXPOSURE**								
**ADULT POPULATIONS**								
**INTERVENTION EVIDENCE**								
1.	Stansfeld et al, Noise Health, 2009	**Population**: adult residents living in noisy and quiet areas, total n=387, aged 16-90yrs. Response rate at baseline 70%, at follow-up 74% **Longitudinal /Intervention** study, baseline in 1997, n=337, follow-up in 1998, n=228 after the opening of a bypass road	**Noise exposure**: road traffic noise levels **measured** between 10 am and 5 pm on weekdays; **Noise source**: road traffic **Noise metrics**: Leq, L10 (dBA) **Noise groups**: noise exposed group (facing the main street, L_{10}=75-78 dBA), control group (on uncongested side streets, L_{10}=55-58	**Comparator**: Noise exposed group compared to control group; within-subjects comparison	**Confounding**: Deprivation index (Townsend's scale including: car ownership, home ownership, crowding, unemployment), baseline health, age, sex	**Outcomes:** **Health status** – SF-36 General Health Survey (Ware & Sherbourne 1992); **Psychological distress** – General Health Questionnaire (GHQ), 28 items (Goldberg & Hillier, 1979); **Prevalence of mental disorders** – assessed by the Revised Clinical Interview	**Findings:** At baseline, people from **high and low noise groups** had similar health status. At follow-up no differences in health status between the groups. At follow-up, after the bypass opened, there was a decrease in prevalence rates of mental disorder in both groups. **Type of analyses:** Univariate analyses of covariance	**Comments:** **Findings:** At follow-up, there was a decrease in noise annoyance in the high noise group.

		(change of noise level by 2-4 dBA)	dBA)			Schedule (Lewis et al, 1992); **Noise annoyance** – from neighbors, road traffic and train noise (Fields, 1992)	(ANCOVA), Univariate analyses of covariance adjusted for baseline health status **Sample size relating to the effect size:** n=387 with complete data; n=71 interviewed for prevalence of mental disorder	
**CROSS-SECTIONAL EVIDENCE**								
2.	Kishikawa, et al, Noise Health, 2009	**Population**: adult residents living along truck roads, total n=323, aged 20-70yrs. **Selection**: n=486 approached, response rate 88.2%, exclusion: aged over 70yrs. **Cross sectional** study	**Noise exposure**: road traffic noise levels at home **measured** in the area for 24 hours; **Noise source**: traffic on the trunk roads **Noise metrics**: Ldn (dBA) **Noise groups**: <55 dBA, 55-65 dBA, >65 dBA	**Comparator**: lower levels of noise exposure	**Confounding**: Age, gender, socioeconomic status, noise sensitivity (sensitive vs. insensitive)	**Outcomes: Subjective health** – using General Health Questionnaire GHQ-28 (Goldberg, 1978) with four subscales (somatic symptoms, anxiety & insomnia, social dysfunction, severe depression); **Noise sensitivity** – using original WNS scale (Weinstein, 1978), and a modified scale (WNS-6B); **Disturbances of daily life** – sleep disturbance, speech	**Findings:** Among noise sensitive persons, the odds ratios for overall GHQ scale, somatic symptoms and anxiety and insomnia increased significantly with Ldn. Among noise-insensitive persons the odds ratio for social dysfunction decreased with Ldn. **Type of analyses:** Multiple logistic regression models **Sample size relating to the effect size:** n=323 with complete data	**Comments: Findings:** Among noise-sensitive persons in the highest noise exposure group (>65 dBA), the odds ratio for psychiatric cases on GHQ scale increased with sleep disturbance – sleep disturbance a possible modifier between noise exposure, sensitivity and psychiatric cases.

						interference, disturbance of watching TV (all assessed using a 5-point scale)		
3.	Honold et al, J Environ Psychol, 2012	**Population**: adult residents living in high noise, high air pollution area, total n=428, aged 16-91yrs. **Selection**: n=2000 approached, n=428 returned, response rate 21.4% **Cross sectional** study	**Noise exposure**: road traffic noise levels **modeled** in the area; **Noise source**: air traffic **Noise metrics**: not specified, probably Leq,24h (dBA) **Noise groups**: High-burden blocks (high noise >65 dBA, high air pollution), low-burden blocks (noise ≤50 dBA)	**Comparator:** High-burden group compared to low-burden group	**Confounding**: Gender, age, educational attainment, nationality, marital status, employment, occupational position, size of community, use of medications, housing conditions	**Outcomes: Neighborhood satisfaction** – using a 4-item scale (Feuersenger, 2004); **Life satisfaction** – assessed using an 5-item scale (Diener et al, 1985); **Health behavior** – smoking, alcohol intake, physical activity (Feldman & Steptoe, 2004); **General physical health** – one item from the SF-36 health survey (Bullinger & Kirchberger, 1998); **Depression and anxiety** – items on the Brief Symptom Inventory (Franke, 2000); **Environmental perception** – six factors, 5-point	**Findings:** People from **high burden blocks** had significantly lower neighborhood satisfaction, and poorer health behavior. Poor health behavior was related to employment, perceived traffic noise and perceived air quality. **Type of analyses:** Analysis of variance (ANOVA), logistic regression, analysis of covariance (ANCOVA), hierarchical regression analysis **Sample size relating to the effect size:** n=215 from high-burden group, n=213 from low-burden group	**Comments: Findings:** No difference in life satisfaction, general physical health, anxiety and depression between high-burden and low-burden groups. **Perceived air quality** was related to life satisfaction, general health, anxiety and depression.

						scale; **Environmental stress appraisal /annoyance** – four factors, 5-point scale		
4.	Sygna et al, Environ Res, 2014	**Population**: adult residents living in one city, total n=2898, aged 18-88yrs. **Selection**: n=5390 eligible, n=3262 returned the questionnaire, response rate 60.5% **Cross sectional** study	**Noise exposure**: road traffic noise levels at the most exposed façade were obtained from official **noise maps** **Noise source**: road traffic **Noise metrics**: Lden (dBA) **Noise groups**: exposed to road traffic noise	**Comparator**: Lower noise levels	**Confounding**: Age, gender, socioeconomic status (income, education, employment), somatic diseases, noise sensitivity, sleep quality categories	**Outcomes: Psychological distress** – measured by Hopkins Symptom Checklist-25 (HSCL- 25) (Derogatis et al, 1974, Strand et al, 2003); **probable mental disorder** defined when mean score ≥1.55; **Sleep quality** – self-reported; **Noise sensitivity** – modified Weinstein's scale (Weinstein, 1978)	**Findings:** In the group with poor sleep quality, the mean score of psychological distress increased by 0.08 per 10 dBA increase in **road traffic** noise exposure (unadjusted model). In the group with poor sleep quality, the odds for probable mental disorder increased by 47% per 10 dBA increase of Lden (unadjusted). **Type of analyses:** Linear regression, logistic regression **Sample size relating to the effect size:** n=2774 with complete data, n=274 with poor sleep quality	**Comments: Findings:** In the groups with medium and good sleep quality, there was no association between noise exposure and the mean score of psychological distress or the odds for probable mental disorder. **Conclusion:** Sleep quality may be modify the relationship between noise and mental health.

Supplementary Table 4: Data extraction for environmental noise effects on interviewer assessed depression and anxiety

	Reference	**Population**: general population in settings (hospitals, residences, public venues, educational facilities) + **response rate** and other **selection** /bias factors **Cross-sectional or longitudinal**	**Exposure**: exposure to high levels of environmental noise from various noise sources + noise metric involved + modelled or measured noise	**Comparator**: no noise exposure or lower levels of noise exposure	**Confounding**: adjusted for confounding	**Outcome**: assessment of outcome	**Findings:** expressed as effect per dB if possible.<br><br>Type of analyses<br><br>Sample size relating to the effect size	**Comments:** Anything else to note
**ROAD NOISE EXPOSURE**								
**ADULT POPULATIONS**								
**INTERVENTION EVIDENCE**								
1.	Stansfeld et al, Noise Health, 2009	**Population**: adult residents living in noisy and quiet areas, total n=387, aged 16-90yrs. Response rate at baseline 70%, at follow-up 74% **Longitudinal /Intervention** study, baseline in 1997, n=337, follow-up in 1998, n=228 after the opening of a bypass road	**Noise exposure**: road traffic noise levels **measured** between 10 am and 5 pm on weekdays; **Noise source**: road traffic **Noise metrics**: Leq, L10 (dBA) **Noise groups**: noise exposed group (facing the main street, L_{10}=75-78 dBA), control group (on uncongested side streets, L_{10}=55-58	**Comparator**: Noise exposed group compared to control group; within-subjects comparison	**Confounding**: Deprivation index (Townsend's scale including: car ownership, home ownership, crowding, unemployment), baseline health, age, sex	**Outcomes:** **Health status** – SF-36 General Health Survey (Ware & Sherbourne 1992); **Psychological distress** – General Health Questionnaire (GHQ), 28 items (Goldberg & Hillier, 1979); **Prevalence of mental disorders** – assessed by the Revised Clinical Interview	**Findings:** At baseline, people from **high and low noise groups** had similar health status. At follow-up no differences in health status between the groups. At follow-up, after the bypass opened, there was a decrease in prevalence rates of mental disorder in both groups. **Type of analyses:** Univariate analyses of covariance	**Comments:** **Findings:** At follow-up, there was a decrease in noise annoyance in the high noise group.

		(change of noise level by 2-4 dBA)	dBA)			Schedule (Lewis et al, 1992); **Noise annoyance** – from neighbors, road traffic and train noise (Fields, 1992)	(ANCOVA), Univariate analyses of covariance adjusted for baseline health status **Sample size relating to the effect size:** n=387 with complete data; n=71 interviewed for prevalence of mental disorder	
**AIRCRAFT NOISE EXPOSURE**								
**CROSS-SECTIONAL EVIDENCE**								
2.	Hardoy et al, Soc Psychiatry Psychiatr Epidemiol, 2005	**Population**: adult residents living in a suburban estate near an airport and quiet districts, total n=111 eligible from exposed area, n=1040 eligible from control area, aged over 18yrs. **Selection**: Control group matched for sex, age, employment **Cross sectional** study	**Noise exposure**: not explained **Noise source:** aircraft noise **Noise metric**: not specified	**Comparator:** Aircraft noise-exposed group compared to quiet area	**Confounding:** Not specified	**Outcomes:** **Life-time prevalence of psychiatric disorders** – assessed by a psychiatric interview, including Generalized Anxiety Disorder (GAD), Anxiety Disorder not otherwise specified (NOS), Major Depressive Disorder, Eating Disorder	**Findings:** People from **aircraft-exposed** area had higher lifetime prevalence of GAD and NOS in comparison to persons from quiet areas. **Type of analyses:** Univariate analysis **Sample size relating to the effect size:** n=71 from aircraft noise exposed group, n=284 from quiet area	**Comments:** The method is observational, no correlation with noise exposure.

Supplementary Table 5: Data extraction for associations of environmental noise exposure and emotional and conduct disorders in children

	Reference	**Population**: general population in settings (hospitals, residences, public venues, educational facilities) + **response rate** and other **selection** /bias factors **Cross-sectional or longitudinal**	**Exposure**: exposure to high levels of environmental noise from various noise sources + noise metric involved + modelled or measured noise	**Comparator**: no noise exposure or lower levels of noise exposure	**Confounding**: adjusted for confounding	**Outcome**: assessment of outcome	**Findings:** expressed as effect per dB if possible.<br><br>Type of analyses<br><br>Sample size relating to the effect size	**Comments:** Anything else to note
**AIRCRAFT NOISE EXPOSURE**								
**CHILD POPULATIONS**								
**CROSS-SECTIONAL EVIDENCE**								
1.	Clark et al, Am J Epidemol, 2012	**Population**: school children, total n=960, aged 9-10yrs. **Cross sectional** study	**Noise exposure**: aircraft noise levels at home and at school **measured** in an area from 7 am to 11 pm; road traffic noise at school **combined** from measurements and models **Noise source**: aircraft and road traffic **Noise metrics**: LAeq, 16h (dB)	**Comparator**: lower levels of noise exposure	**Confounding**: Modelled concentrations of **NO2** (µg/m3); age, gender, socio-economic status (employment, home ownership, home crowding), ethnicity, maternal education, main language spoken at home, long-standing illness, parental support for schoolwork, classroom glazing	**Outcome**: **Perceived child's health** – very good/good vs. fair/poor/very poor, perceived by parents; **Psychological distress** measured using Strengths and Difficulties Questionnaire (Goodman, 1997), fulfilled by parents	**Findings:** No association between aircraft and road traffic noise and self-rated health or psychological distress **Type of analyses:** Multilevel linear and logistic regression models **Sample size relating to the effect size:** n=719 with air pollution data and n=241 without air pollution data	**Comments:** **Findings:** No association between aircraft and road traffic noise and systolic and diastolic blood pressure
2.	Clark et al, J	**Population**:	**Noise exposure**:	**Compared**	**Confounding**:	**Outcome**:	**Findings:**	**Comments:**

	Enviro Psychol, 2013	school children, total n=461, aged 15-16yrs. **Sampling procedure**: baseline sample tested in 2001-2003: 1355 children aged 9-10yrs; follow-up sample testing in 2008: 1015 children eligible for testing, 461 children participated, aged 14-15; response rate 45.4% **Longitudinal study;** follow-up period: 6 years (2001/2003-2008)	aircraft noise levels at primary and secondary schools **measured** in an area from 7 am to 11 pm; road traffic noise at school **combined** from measurements and models available for elementary schools **Noise source**: aircraft **Noise metrics**: LAeq, 16h (dB)	**to**: noise levels at secondary schools compared to noise levels at primary schools	age, gender, employment, home ownership, home crowding, mother's educational attainment, long-standing illness, parental support for school work at baseline, classroom glazing at primary school, road traffic noise	**Psychological distress** (including emotional symptoms, conduct problems and hyperactivity) was measured using Strengths and Difficulties Questionnaire (Goodman, 1997), fulfilled by parents at baseline, fulfilled by children at follow-up; **Aircraft noise annoyance** measured by 5-point scale (ISO 2003) at baseline and at follow-up fulfilled by children	Increase of aircraft noise at secondary school by 1 dB was non-significantly associated with a decrease in psychological distress by 0.023 marks, and in emotional symptoms by 0.034 points; and with an increase of hyperactivity by 0.001 marks, and of conduct problems by 0.011 marks; Increase of aircraft noise at secondary school by 1 dB was significantly associated with an increase of noise annoyance by 0.048 marks (unadjusted), or 0.043 marks (adjusted) **Type of analyses:** Multilevel linear regression analyses **Sample size relating to the effect size:** n=461 with complete data	**Findings:** Increase of aircraft noise at primary school or of cumulative aircraft noise at school by 1 dB were significantly associated with an increase of aircraft noise annoyance
3.	Crombie et al, Enviro Health, 2011	**Population**: school children, 89 schools around three airports, total n=2279, aged 10yrs. Response rate: 89%	**Noise exposure**: aircraft noise levels at school **measured** in an area from 7 am to 11 pm; road traffic noise at school **modelled** or **combination**	**Compared to**: lower levels of noise exposure	**Confounding**: early biological risk (low birth weight or born prematurely); country, gender, age, employment status, crowding at home, home ownership,	**Outcome**: **Mental health** was measured using Strengths and Difficulties Questionnaire (subscales: emotional symptoms,	**Findings:** Increase of **aircraft** noise at school by 1 dB was significantly associated with an increase of hyperactivity by 0.01 points (adjusted for all confounders, except biological risk).	**Comments: Findings:** Aircraft noise at school was not associated with overall mental health score, emotional symptoms, and

		**Cross sectional** study	of measurements with models<br>**Noise source**: aircraft and road traffic<br>**Noise metrics**: LAeq, 16h (dB)		mother's education, long-standing illness, main language spoken at home, parental support for school work, classroom glazing	conduct problems and hyperactivity) (Goodman, 1997), fulfilled by parents;	Increase of **road traffic** noise at school by 1 dB was significantly associated with an increase of conduct problems by 0.01 points (fully adjusted).<br>**Type of analyses:** Multilevel linear regression analyses<br>**Sample size relating to the effect size:** n=1900 with complete data	conduct problems (either unadjusted or adjusted for early biological risk).<br>Road traffic noise at school was not associated with overall mental health score, emotional symptoms, and hyperactivity (either unadjusted or adjusted for early biological risk).<br>No interaction between noise exposure and early biological risk.
4.	Stansfeld et al, J Enviro Psychol, 2009	**Population**: school children, 89 schools around three airports, total n=2844, aged 9-10yrs. Response rate 89% among children, 80% among parents.<br>**Cross sectional** study	**Noise exposure**: aircraft noise levels at home and at school **measured** in an area from 7 am to 11 pm; road traffic noise at school **modelled** or **combination** of measurements with models<br>**Noise source**:	**Comparator**: lower levels of noise exposure	**Confounding**: age, gender, country, socio-economic position – occupation, employment, free meal at school, maternal education, ethnicity, main language spoken at home; home ownership, crowding, long-standing illness,	**Outcomes:**<br>**Mental health** measured using Strengths and Difficulties Questionnaire (Goodman, 1997), fulfilled by parents, subscales: hyperactivity, conduct disorder, peer problems, prosocial	**Findings:**<br>Increase of **aircraft** noise at school by 1 dB was significantly associated with an increase of hyperactivity score by 0.013 points.<br>Increase of **road-traffic** noise at school by 1 dB was significantly associated with a decrease of conduct disorder by 0.010 points.<br>**Type of analyses:**	**Comments:**<br>**Findings:**<br>No association between aircraft or road traffic noise and children's mental health (total SDQ score).

			aircraft and road traffic<br>**Noise metrics**: LAeq, 16h (dB)		parental support, classroom glazing, other noise exposure	behavior, emotional problems;<br>**Attention-Deficit Hyperactivity Disorder** (ADHD) – assessed by parental questionnaire	Multilevel model analyses<br>**Sample size relating to the effect size:** n=2844	
5.	Stansfeld et al, Lancet, 2005	**Population**: school children, 89 schools around three airports, total n=2844, aged 9-13yrs.<br>**Cross sectional** study	**Noise exposure**: aircraft noise levels at home and at school **measured** in an area from 7 am to 11 pm; road traffic noise at school **modelled** or **combination** of measurements with models<br>**Noise source**: aircraft and road traffic<br>**Noise metrics**: LAeq, 16h (dB)	**Comparator**: lower levels of noise exposure	**Confounding**: age, gender, parental employment, home ownership, home crowding, mother's educational attainment, long-standing illness, main language spoken at home, parental support for school work, classroom glazing	**Outcomes:**<br>**Self-reported health** – perceived by children;<br>**Psychological distress** – perceived children's mental health, measured using Strengths and Difficulties Questionnaire (Goodman, 1997), fulfilled by parents<br>**Aircraft noise annoyance** measured by 5-point scale (ISO 2003) fulfilled by children	**Findings:**<br>Increase of **aircraft** noise at school by 1 dB was significantly associated with an increase of noise annoyance by 0.037 marks.<br>Increase of **road traffic** noise at school by 1 dB was significantly associated with an increase of noise annoyance by 0.016 marks.<br>**Type of analyses:** Multilevel model analyses (for data clustering)<br>**Sample size relating to the effect size:** range from 1939 to 2014 with complete data	**Comments:**<br>**Findings:**<br>No association between aircraft and road traffic noise and self-reported health, and perceived children's mental health.
**ROAD TRAFFIC NOISE**								
**LONGITUDINAL EVIDENCE**								
6.	Hjorteberg et al, Env Health	**Population**: a total of 46.940	**Noise exposure**: noise levels	**Comparison:** Children	**Confounding:** Age, sex,	**Outcomes:**<br>**Behavioral**	**Findings:**<br>An increase of Lden per	**Comments:**<br>No association

	Perspect, 2015	7-year old children **Selection:** Inclusion: only children from first pregnancy were included out of 57,281 eligible children; Exclusion: missing data **Study design:** national **birth cohort**	were **modeled** **Noise source**: road traffic and railway noise **Noise metrics**: Lden (dBA) **Noise exposure intervals:** 1) during pregnancy, and 2) from birth to 7 years	exposed to higher noise levels compared to children exposed to lower noise levels	gestational age, birth weight, maternal age at delivery, parity, smoking during pregnancy, alcohol during pregnancy, education level, income, railway and airport noise, maternal mental health problems, time-weighted NOx level	**problems** – measured with Strengths and Difficulties Questionnaire (SDQ-Danish version, Goodman, 1997; Obel et al, 2003); subscales: emotional symptoms, conduct problems, hyperactivity/ inattention, peer relationship problems; fulfilled by mother; scores were classified as normal/ borderline/ abnormal (Danish cut-off scores, Youthin Mind, 2015)	10 dBA for **road-traffic** noise from birth to 7 years significantly increases odds ratios for abnormal total scores, for borderline and abnormal hyperactivity/ inattention scores, for abnormal conduct problem scores and for abnormal peer relationship scores. An increase of Lden per 10 dBA for **railway** noise from birth to 7 years significantly increases ORs for abnormal total scores, and for abnormal peer relationship scores. **Type of analyses:** Multinomial logistic regression, logistic regression **Sample size relating to the effect size:** n=46940 for road traffic exposure; n=3770 for railway exposure	between exposure to road traffic /railway noise during pregnancy and behavioral problems. No significant effect modification by sec, low birth weight, educational level, income.
**CROSS-SECTIONAL EVIDENCE**								

7.	Tiesler et al, Enviro Res, 2013	**Population**: two birth cohorts, school children, total n=872, aged 10yrs.<br>**Inclusion criteria**: participation at 10-year follow up of the cohort, available noise exposure data, available data on behavioral problems;<br>Exclusion: living less than 1yr at given address<br>**Cross sectional** study	**Noise exposure**: road traffic noise levels at home and at school **modelled** from noise maps<br>**Noise source**: road traffic<br>**Noise metrics**: Lden (dBA), Lnight (from 10 p.m. to 6 a.m.) (dBA) at the most and the least exposed façade	**Comparator**: Interquartile range of noise levels	**Confounding**: Age, sex, study group, parental education, television/computer usage, mother's age at child's birth, single parent status; sleeping alone in the room, bedroom window orientation	**Outcomes:**<br>**Behavioral problems** – measured with Strengths and Difficulties Questionnaire (SDQ, Goodman, 1997), (subscales: emotional symptoms, conduct problems, hyperactivity/ inattention, peer relationship problems) fulfilled by parents;<br>**Sleep problems** – including difficulties falling asleep, problems sleeping through the night	**Findings:**<br>An increase of Lden and of Lnight (per IQR) at the most exposed façade significantly increased odds ratios for hyperactivity/inattention (crude and adjusted).<br>An increase of Lden and of Lnight (per IQR) at the least exposed façade significantly increased odds ratios for abnormal emotional symptoms (crude and adjusted).<br>Lden and Lnight were not associated with overall behavioral problems (total SDQ score).<br>**Type of analyses:**<br>Logistic regression analyses, continuation ration models<br>**Sample size relating to the effect size:**<br>n=872 with complete data, n=287 with complete data on sleep problems	**Comments:**<br>**Findings:**<br>No association between Lnight at the most exposed façade and sleep problems.<br>Lnight at the least exposed façade associated with any sleeping problems and problems falling asleep (but not problems during sleeping) (crude and adjusted).
8	Belojevic, et al, J Environ Psychol, 2012	**Population**: urban school children from eight schools, total n=311, aged 7-11yrs.<br>**Sampling**	**Noise exposure**: noise levels **measured** outside schools (from 9 am to 5 pm, Leq 8h) and in the streets of	**Compared to:** lower levels of noise exposure	**Confounding**: Age, gender, socio-economic score – obtained as a sum of standardized mother's highest education level and	**Outcome**:<br>**Executive functioning** (defined as decision making, working memory and self-	**Findings:**<br>All **road-traffic** noise parameters at home (daytime Leq, nighttime Leq, Leq 24 h, traffic density) were negatively correlated to mean	**Comments:**<br>**Findings**:<br>No correlation between noise metrics at school; no effect on girls.

		**procedure**: 2000 children approached, 1150 parents granted permission to participate; some teachers refused to complete questionnaire. **Exclusion criterion**: living outside the selected municipality **Cross sectional** study	children's residences (Leq 24h); traffic density – number of light and heavy vehicles counted in all streets **Noise source**: road traffic **Noise metrics at home**: daytime Leq (dBA), nighttime Leq (dBA), Leq 24h (dBA), light vehicles at daytime and at night (N/hour), heavy vehicles at daytime and at night (N/hour) **Noise metrics at school**: daytime Leq (dBA); light vehicles at daytime (N/hour), heavy vehicles at daytime (N/hour)		standardized income variable	regulation of emotions and behaviors) – scale adapted from Attention Deficit Disorder Questionnaire (Sears and Thompson, 1998), children's functioning was assessed by teachers rated	executive functioning score in boys; **Road-traffic** Leq 24h at home significantly related to executive functioning after adjustment for socio-economic status in models. **Type of analyses**: Correlation coefficients (Pearson's, Spearman's); Multiple linear regression analysis (Leq 24h at home as continuous variable) **Sample size relating to the effect size:** n=146 boys	
	See Clark et al, Am J Epidemol, 2012							
	See Crombie et al, Enviro							

	Health, 2011							
	See Stansfeld et al, J Enviro Psychol, 2009							
	See Stansfeld et al, Lancet, 2005							
**RAILWAY NOISE EXPOSURE**								
**LONGITUDINAL EVIDENCE**								
	See Hjorteberg et al, Env Health Perspect, 2015 above							

Supplementary Table 6: Data extraction for associations of environmental noise exposure and hyperactivity in children

	Reference	**Population**: general population in settings (hospitals, residences, public venues, educational facilities) + **response rate** and other **selection** /bias factors **Cross-sectional or longitudinal**	**Exposure**: exposure to high levels of environmental noise from various noise sources + noise metric involved + modelled or measured noise	**Comparator**: no noise exposure or lower levels of noise exposure	**Confounding**: adjusted for confounding	**Outcome**: assessment of outcome	**Findings:** expressed as effect per dB if possible.<br><br>Type of analyses<br><br>Sample size relating to the effect size	**Comments:** Anything else to note
**AIRCRAFT NOISE EXPOSURE**								
**CHILD POPULATIONS**								
**CROSS-SECTIONAL EVIDENCE**								
1.	Clark et al, J Enviro Psychol, 2013	**Population**: school children, total n=461, aged 15-16yrs. **Sampling procedure**: baseline sample tested in 2001-2003: 1355 children aged 9-10yrs; follow-up sample testing in 2008: 1015 children eligible for testing, 461 children participated, aged 14-15; response rate 45.4%	**Noise exposure**: aircraft noise levels at primary and secondary schools **measured** in an area from 7 am to 11 pm; road traffic noise at school **combined** from measurements and models available for elementary schools **Noise source**: aircraft **Noise metrics**: LAeq, 16h (dB)	**Compared to**: noise levels at secondary schools compared to noise levels at primary schools	**Confounding**: age, gender, employment, home ownership, home crowding, mother's educational attainment, long-standing illness, parental support for school work at baseline, classroom glazing at primary school, road traffic noise	**Outcome**: **Psychological distress** (including emotional symptoms, conduct problems and hyperactivity) was measured using Strengths and Difficulties Questionnaire (Goodman, 1997), fulfilled by parents at baseline, fulfilled by children at follow-up; **Aircraft noise annoyance** measured by 5-point scale (ISO 2003) at baseline and at follow-up fulfilled by children	**Findings:** Increase of aircraft noise at secondary school by 1 dB was non-significantly associated with a decrease in psychological distress by 0.023 marks, and in emotional symptoms by 0.034 points; and with an increase of hyperactivity by 0.001 marks, and of conduct problems by 0.011 marks; Increase of aircraft noise at secondary school by 1 dB was significantly associated with an increase of noise annoyance by 0.048	**Comments:** **Findings:** Increase of aircraft noise at primary school or of cumulative aircraft noise at school by 1 dB were significantly associated with an increase of aircraft noise annoyance

		**Longitudinal study;** follow-up period: 6 years (2001/2003-2008)					marks (unadjusted), or 0.043 marks (adjusted)<br>**Type of analyses:** Multilevel linear regression analyses<br>**Sample size relating to the effect size:** n=461 with complete data	
2.	Crombie et al, Enviro Health, 2011	**Population**: school children, 89 schools around three airports, total n=2279, aged 10yrs.<br>Response rate: 89%<br>**Cross sectional** study	**Noise exposure**: aircraft noise levels at school **measured** in an area from 7 am to 11 pm; road traffic noise at school **modelled** or **combination** of measurements with models<br>**Noise source**: aircraft and road traffic<br>**Noise metrics**: LAeq, 16h (dB)	**Compared to**: lower levels of noise exposure	**Confounding**: early biological risk (low birth weight or born prematurely); country, gender, age, employment status, crowding at home, home ownership, mother's education, long-standing illness, main language<br>spoken at home, parental support for school work, classroom glazing	**Outcome**: **Mental health** was measured using Strengths and Difficulties Questionnaire (subscales: emotional symptoms, conduct problems and hyperactivity) (Goodman, 1997), fulfilled by parents;	**Findings:** Increase of **aircraft** noise at school by 1 dB was significantly associated with an increase of hyperactivity by 0.01 points (adjusted for all confounders, except biological risk). Increase of **road traffic** noise at school by 1 dB was significantly associated with an increase of conduct problems by 0.01 points (fully adjusted).<br>**Type of analyses:** Multilevel linear regression analyses<br>**Sample size relating to the effect size:** n=1900 with complete data	**Comments:**<br>**Findings:** Aircraft noise at school was not associated with overall mental health score, emotional symptoms, and conduct problems (either unadjusted or adjusted for early biological risk).<br>Road traffic noise at school was not associated with overall mental health score, emotional symptoms, and hyperactivity (either unadjusted or adjusted for early biological risk).<br>No interaction between noise exposure and

								early biological risk.
3.	Stansfeld et al, J Enviro Psychol, 2009	**Population**: school children, 89 schools around three airports, total n=2844, aged 9-10yrs. Response rate 89% among children, 80% among parents. **Cross sectional** study	**Noise exposure**: aircraft noise levels at home and at school **measured** in an area from 7 am to 11 pm; road traffic noise at school **modelled** or **combination** of measurements with models **Noise source**: aircraft and road traffic **Noise metrics**: LAeq, 16h (dB)	**Comparator**: lower levels of noise exposure	**Confounding**: age, gender, country, socio-economic position – occupation, employment, free meal at school, maternal education, ethnicity, main language spoken at home; home ownership, crowding, long-standing illness, parental support, classroom glazing, other noise exposure	**Outcomes: Mental health** measured using Strengths and Difficulties Questionnaire (Goodman, 1997), fulfilled by parents, subscales: hyperactivity, conduct disorder, peer problems, prosocial behavior, emotional problems; **Attention-Deficit Hyperactivity Disorder** (ADHD) – assessed by parental questionnaire	**Findings:** Increase of **aircraft** noise at school by 1 dB was significantly associated with an increase of hyperactivity score by 0.013 points. Increase of **road-traffic** noise at school by 1 dB was significantly associated with a decrease of conduct disorder by 0.010 points. **Type of analyses:** Multilevel model analyses **Sample size relating to the effect size:** n=2844	**Comments: Findings:** No association between aircraft or road traffic noise and children's mental health (total SDQ score).
**ROAD TRAFFIC NOISE**								
**LONGITUDINAL EVIDENCE**								
4.	Hjorteberg et al, Env Health Perspect, 2015	**Population**: a total of 46.940 7-year old children **Selection:** Inclusion: only children from first pregnancy were included out of 57,281 eligible children; Exclusion: missing data **Study design:** national **birth cohort**	**Noise exposure**: noise levels were **modeled** **Noise source**: road traffic and railway noise **Noise metrics**: Lden (dBA) **Noise exposure intervals:** 1) during pregnancy, and 2) from birth to 7 years	**Comparison:** Children exposed to higher noise levels compared to children exposed to lower noise levels	**Confounding:** Age, sex, gestational age, birth weight, maternal age at delivery, parity, smoking during pregnancy, alcohol during pregnancy, education level, income, railway and airport noise, maternal mental health problems, time-weighted NOx level	**Outcomes: Behavioral problems** – measured with the hyperactivity/inattention sub-scale from the Strengths and Difficulties Questionnaire (SDQ-Danish version, Goodman, 1997; Obel et al, 2003). This was completed y mother; scores were classified as normal/ borderline/ abnormal (Danish cut-off scores, Youthin Mind, 2015)	**Findings:** An increase of Lden per 10 dBA for **road-traffic** noise from birth to 7 years significantly increases odds ratios for abnormal hyperactivity/ inattention scores **Type of analyses:** Multinomial logistic regression, logistic regression **Sample size relating to the effect size:** n=46940 for road traffic exposure; n=3770 for railway exposure	**Comments:** No association between exposure to road traffic /railway noise during pregnancy and hyperactivity problems. No significant effect modification by sec, low birth weight, educational level, income.
**CROSS-SECTIONAL EVIDENCE**								

5.	Tiesler et al, Enviro Res, 2013	**Population**: two birth cohorts, school children, total n=872, aged 10yrs.<br>**Inclusion criteria**: participation at 10-year follow up of the cohort, available noise exposure data, available data on behavioral problems;<br>Exclusion: living less than 1yr at given address<br>**Cross sectional** study	**Noise exposure**: road traffic noise levels at home and at school **modelled** from noise maps<br>**Noise source**: road traffic<br>**Noise metrics**: Lden (dBA), Lnight (from 10 p.m. to 6 a.m.) (dBA) at the most and the least exposed façade	**Comparator**: Interquartile range of noise levels	**Confounding**: Age, sex, study group, parental education, television/computer usage, mother's age at child's birth, single parent status; sleeping alone in the room, bedroom window orientation	**Outcomes:**<br>**Behavioral problems** – measured with Strengths and Difficulties Questionnaire (SDQ, Goodman, 1997), (subscales: emotional symptoms, conduct problems, hyperactivity/ inattention, peer relationship problems) fulfilled by parents;<br>**Sleep problems** – including difficulties falling asleep, problems sleeping through the night	**Findings:**<br>An increase of Lden and of Lnight (per IQR) at the most exposed façade significantly increased odds ratios for hyperactivity/inattention (crude and adjusted).<br>An increase of Lden and of Lnight (per IQR) at the least exposed façade significantly increased odds ratios for abnormal emotional symptoms (crude and adjusted).<br>Lden and Lnight were not associated with overall behavioral problems (total SDQ score).<br>**Type of analyses:**<br>Logistic regression analyses, continuation ration models<br>**Sample size relating to the effect size:**<br>n=872 with complete data, n=287 with complete data on sleep problems	**Comments:**<br>**Findings:**<br>No association between Lnight at the most exposed façade and sleep problems.<br>Lnight at the least exposed façade associated with any sleeping problems and problems falling asleep (but not problems during sleeping) (crude and adjusted).
	See Crombie et al, Enviro Health, 2011							
	See Stansfeld et al, J Enviro Psychol, 2009							

Supplementary Table 7: Data extraction for associations of environmental noise exposure and other mental health outcomes (not falling into any of the above outcome domains)

	Reference	**Population**: general population in settings (hospitals, residences, public venues, educational facilities) + **response rate** and other **selection** /bias factors **Cross-sectional or longitudinal**	**Exposure**: exposure to high levels of environmental noise from various noise sources + noise metric involved + modelled or measured noise	**Comparator**: no noise exposure or lower levels of noise exposure	**Confounding**: adjusted for confounding	**Outcome**: assessment of outcome	**Findings:** expressed as effect per dB if possible.<br><br>Type of analyses<br><br>Sample size relating to the effect size	**Comments:** Anything else to note
**ROAD TRAFFIC NOISE EXPOSURE**								
**ADULT POPULATIONS**								
**CROSS-SECTIONAL EVIDENCE**								
1.	Fooladi, J Environ Public Health, 2012	**Population**: adult residents living and working in a single noisy street, total n=83, aged 18-38yrs. **Inclusion criteria**: living in the area for 6 months, language	**Noise exposure**: road traffic noise levels **measured** near home and workplace **Noise source**: road traffic **Noise metrics**: L at morning, noon, afternoon (dBA)	**Comparator**: None	**Confounding**: Not specified	**Outcomes:** Self-reported **frustration, anger**, and **feeling helpless** as an aftereffect of persistent noise; Self-reported **coping skills** when sharing experiences (craving for	**Findings:** Sleep difficulties reported by 70%; 81% of women reported frustration, anger, feeling helpless as an aftereffect of persistent noise. 93% of men reported habituation techniques; 78% of women reported headaches. **Type of analyses:** none	**Comments:** The method is observational, no comparisons, no correlation with noise exposure.

		literacy, not exposed to loud music<br>**Cross sectional** study				sweets, caffeine intake, smoking);<br>Self-reported techniques for **habituation to noise** (chewing gums, tooth picks, snacking at work, using incessant noise to sleep);<br>Self-reported **sleep disturbance**;<br>Self-reported **headaches**	**Sample size relating to the effect size:** n=83	
2.	Urban & Maca, Int J Environ Res Public Health, 2013	**Population**: adult residents living in six noisy areas, random sampling, total n=354 exposed to road traffic noise, n=228 exposed to railway noise, aged 18-88yrs.<br>**Cross sectional** study	**Noise exposure**: road traffic noise levels obtained from official **noise maps**<br>**Noise source**: road or railway traffic<br>**Noise metrics**: Lden (dBA)<br>**Noise groups**: exposed to road traffic noise (4 areas), exposed to railway noise (2 areas)	**Comparator**: Comparison between the areas exposed to road traffic or railway traffic	**Confounding**: Noise source, noise annoyance	**Outcomes:**<br>**Life satisfaction** – assessed using an 11-point scale (Cantril, 1966);<br>**Residential satisfaction** – measured on an 11-point Likert-type scale;<br>**Noise sensitivity** – answered one question on a 4-point scale;<br>**Noise annoyance** – from road traffic and railway traffic (ISO, 15666:2003)	**Findings:**<br>No difference in the average life satisfaction between 4 areas exposed to road traffic noise / between 2 areas exposed to railway noise.<br>Negative effect of road traffic annoyance on life satisfaction and residential satisfaction.<br>Negative effect of railway annoyance on residential satisfaction.<br>**Type of analyses:** Analysis of variance (ANOVA), structural equation modeling (SEM)<br>**Sample size relating to the effect size:** n=582 with complete data	**Comments:**<br>**Findings:** Proposed models to explain the relationship between noise, noise sensitivity, annoyance, life satisfaction and residential satisfaction.
